# *Alliaceae* versus *Brassicaceae* for Dyslipidemia: State of the Art and Future Perspectives. Systematic Review and Meta‐Analysis of Clinical Studies

**DOI:** 10.1002/ptr.8350

**Published:** 2024-09-29

**Authors:** Eugenia Piragine, Marco Andrea Malanima, Costanza Ceccanti, Lucia Guidi, Alma Martelli, Ersilia Lucenteforte, Vincenzo Calderone

**Affiliations:** ^1^ Department of Pharmacy University of Pisa Pisa Italy; ^2^ Interdepartmental Research Center “Nutraceuticals and Food for Health (NUTRAFOOD)” University of Pisa Pisa Italy; ^3^ Clinical Trial Center Careggi University Hospital Florence Italy; ^4^ Department of Agriculture, Food and Environment University of Pisa Pisa Italy; ^5^ Department of Statistics, Computer Science, Applications “G. Parenti” (DiSIA) University of Florence Florence Italy

**Keywords:** *Alliaceae*, *Brassicaceae*, dyslipidemia, hypercholesterolemia, nutraceuticals, organosulfur compounds

## Abstract

Dyslipidemia is a risk factor for cardiovascular diseases. Preclinical studies have shown that organosulfur compounds from the *Alliaceae* and *Brassicaceae* plants, such as garlic (*Allium sativum* L.) and broccoli (*Brassica oleracea* L.), have potential lipid‐lowering effects. However, their clinical efficacy is controversial, especially in “drug‐free” patients. The aim of this work was to summarize evidence on the lipid‐lowering properties of extracts containing organosulfur compounds in patients with dyslipidemia. Studies were searched in four databases (Medline, Scopus, Embase, and CENTRAL), from inception to October 11, 2023.Controlled clinical studies on patients with dyslipidemia receiving *Alliaceae* or *Brassicaceae* were included. The outcome was the change in lipid parameters from baseline. Random‐effect meta‐analysis of the extracted data was performed using R software. The effect size was expressed as mean difference (MD) and 95% confidence interval (CI). The certainty of evidence was assessed with the GRADE approach. Out of 28 studies that were reviewed, 22 were included in the meta‐analysis (publication period: 1981–2022). Results showed that *Alliaceae* extracts significantly reduce total cholesterol [MD: −15.2 mg/dL; 95% CI: −21.3; −9.1] and low‐density lipoprotein cholesterol levels [MD: −12.0 mg/dL; 95% CI: −18.1; −5.7], although with low certainty of evidence. Conversely, the lipid‐lowering properties of *Brassicaceae* extracts are still unexplored. Our results support the use of *Alliaceae* extracts in patients with hypercholesterolemia, but future high‐quality studies are needed. Our work suggests further exploration of the efficacy of *Brassica*c*eae* extracts, which may have high nutraceutical/phytotherapeutic potential, opening new perspectives in the management of dyslipidemia.

## Graphical abstract

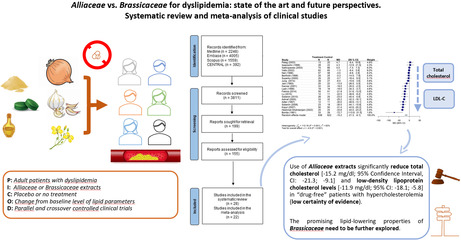

## 1 Introduction

Dyslipidemia is a recognized risk factor for cardiovascular diseases (CVDs) that are the main causes of hospitalization and mortality worldwide (Du and Qin 2023). There is currently no information on the global prevalence of dyslipidemia, but a systematic review on the topic is ongoing (Mohamed‐Yassin et al. 2021). In the United States, the prevalence of lipid disorders in the early 2000s was about 53.0% (Toth, Potter, and Ming 2012); in the United Kingdom, in 2019, it was 23.5% (Bilitou et al. 2022), while in the Asia‐Pacific region in 2021, it ranged from 13.9% to 72.0%, depending on the type of lipid abnormality (Lee et al. 2021). Indeed, the term “dyslipidemia” refers to the imbalance in plasma levels of triglycerides (TGs), total cholesterol (TC), or its fractions low‐density lipoprotein cholesterol (LDL‐C), and high‐density lipoprotein cholesterol (HDL‐C). Therefore, this pathological condition can be divided into three main forms: hypercholesterolemia, hypertriglyceridemia, or a combined pattern (Du and Qin 2023; Thongtang et al. 2022). Among these, hypercholesterolemia is the most common form of dyslipidemia, with a prevalence of 39% in 2008 worldwide, according to the latest update of World Health Organization (WHO) estimates (WHO 2008).

Dyslipidemia can result from genetic predisposition (primary or familial dyslipidemia), external factors (secondary dyslipidemia), or a combination of both (Berberich and Hegele 2019). Among external factors, the main contributors are metabolic disorders (e.g., diabetes or obesity), sedentary lifestyle, and unhealthy diet (i.e., excessive intake of calories, saturated fats, and dietary cholesterol) (Yanai and Yoshida 2021). Over the last years, the global prevalence of dyslipidemia has progressively increased, thus representing a serious threat for the healthcare systems. This trend is particularly evident among young adults (Liu et al. 2023), with an estimated prevalence of 13.0% in Chinese children and adolescents (Liu et al. 2023) and 29.0% in the 18–40 age group of another Chinese cohort (Liu et al. 2018). This could be due to unrecognized familial hypercholesterolemia, unhealthy habits (e.g., sedentary lifestyle, atherogenic diet, smoking, and/or alcohol consumption), or the presence of risk factors, such as overweight/obesity (Schefelker and Peterson 2022), which can have a huge impact on blood TGs, TC, and LDL‐C even in young people (Liu et al. 2023). Alarmingly, high plasma levels of LDL‐C have risen from the 15th risk factor for death to the 8th in less than 20 years (Thongtang et al. 2022), confirming the above trend. The association between high levels of LDL‐C and the development of atherosclerotic cardiovascular disease (ASCVD) (Pirillo et al. 2021) is well established, and it is also known that hypertriglyceridemia enhances the risk of CVDs (Sniderman et al. 2018). Moreover, hypertriglyceridemia is one of the triggers of non‐CVDs, such as acute pancreatitis and nonalcoholic fatty liver disease (Thongtang et al. 2022). Hence, the prevention and treatment of dyslipidemia are fundamental for the prophylaxis of CVDs and multiple associated disorders.

As meticulously reviewed by Aygun and Tokgozoglu in their “recent comparison of current international guidelines for the management of dyslipidemia” (Aygun and Tokgozoglu 2022), the cutoff values for the diagnosis, as well as the criteria for the establishment of risk categories, are slightly divergent among the different guidelines. However, all of them agree on the key role of a healthy diet in the prevention of dyslipidemia and, as first‐line treatment, recommend the use of statins for patients with dysregulated lipid metabolism. Despite their proved clinical efficacy, the regular use of statins can increase the risk of adverse events (Newman et al. 2019) and suboptimal medication adherence/persistence (Maningat, Gordon, and Breslow 2013). Thus, the discovery of new therapeutic options (i.e., lipid‐lowering drugs, foods, or extracts) is a compelling challenge.

**FIGURE 1:**
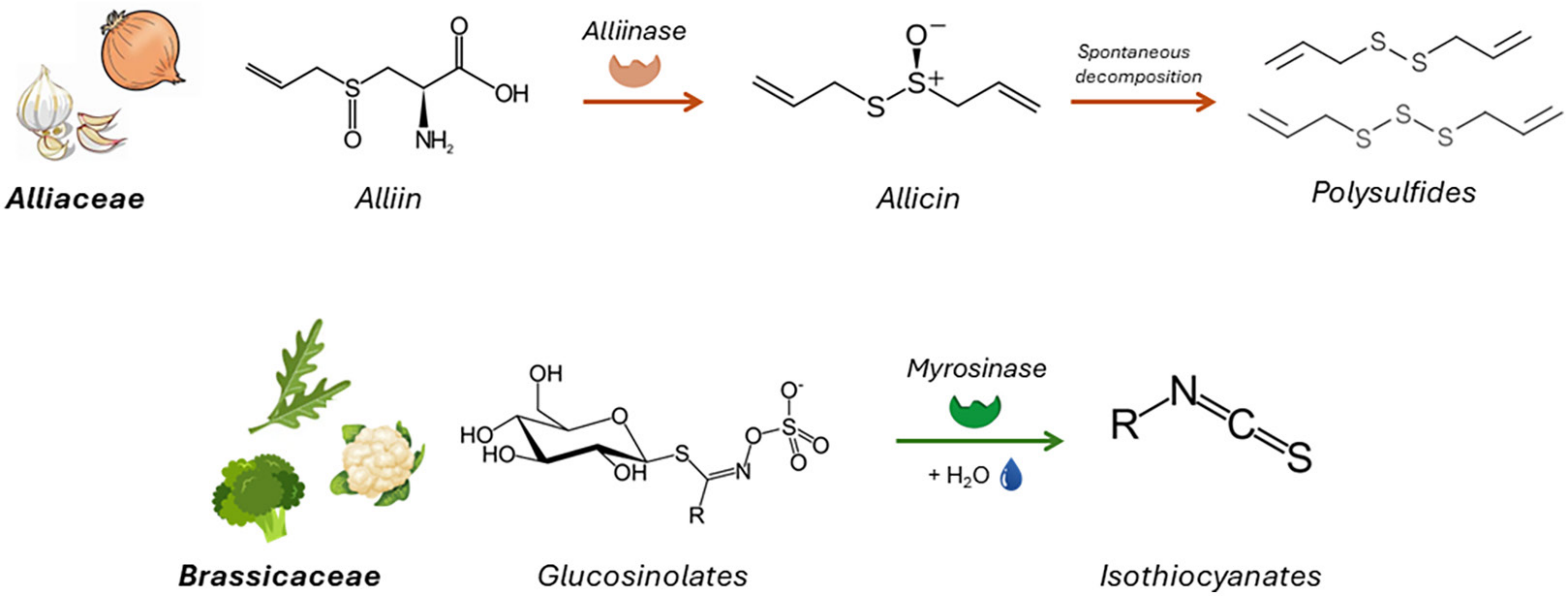
Biosynthesis and chemical structure of polysulfides from *Alliaceae* and isothiocyanates from *Brassicaceae*. Polysulfides result from the conversion of the precursor alliin to allicin, catalyzed by the enzyme alliinase, and subsequent spontaneous decomposition of allicin. Isothiocyanates are the products of the hydrolysis of glucosinolates, operated by the enzyme myrosinase.

In recent times, edible plants from the *Alliaceae* (e.g., onion, *Allium cepa* L.; garlic, *Allium sativum* L.) and *Brassicaceae*/*Cruciferae* (i.e., rocket salad, *Eruca sativa* Mill.; broccoli, *Brassica oleracea* L., etc.) families have gained particular interest. These plants, which have been part of the Mediterranean diet since ancient times, have a well‐documented safety profile and are used as traditional medicine worldwide (Petrovska and Cekovska 2010; Teshika et al. 2019). They contain high levels of organosulfur compounds, recently defined for the first time as “sulfaceutics” (Martelli et al. 2023), which are converted into the active metabolites polysulfides and isothiocyanates upon hydrolysis by plant enzymes (alliinase and myrosinase, respectively) (Lawson and Hunsaker 2018; Narbad and Rossiter 2018). Briefly, polysulfides derive from the precursor alliin, which can be found in plants from the *Alliaceae* family, while isothiocyanates are generated from the hydrolysis of glucosinolates, the precursors present in *Brassica* vegetables (Lawson and Hunsaker 2018; Narbad and Rossiter 2018) **(**Figure 1 **).**

Natural polysulfides and isothiocyanates can generate hydrogen sulfide (H_2_S) (Benavides et al. 2007; Citi et al. 2014), a gasotransmitter whose demonstrated cardiometabolic properties range from cardioprotective and vasoprotective (Citi et al. 2021, 2018; Shen et al. 2015) to antihypertensive and glucose‐lowering (Piragine and Calderone 2021; Piragine et al. 2022b). Interestingly, edible plants containing organosulfur compounds also promote CV and metabolic health. Indeed, they regulate glucose metabolism in patients with type 2 diabetes (T2D) (Piragine, Petri, et al. 2022), decrease blood pressure in subjects with hypertension (Piragine et al. 2022a), and reduce the incidence of CV outcomes in healthy people (Bahadoran et al. 2017; Zhang et al. 2011). The striking overlap in the cardiometabolic effects of H_2_S and edible plants containing organosulfur compounds might partially result from the H_2_S‐releasing behavior of polysulfides and isothiocyanates. Further evidence was provided by the results of recent preclinical studies, which individually demonstrated that the endogenous gasotransmitter H_2_S is an emerging regulator of lipid metabolism (Sun, Wu, et al. 2021) and that *Alliaceae* and *Brassicaceae* extracts promote lipid‐lowering effects in animal models of dyslipidemia (Mohammadi and Oshaghi 2014; Piragine et al. 2021). This property could result in part from the bioactivity of polyphenolic compounds found in plants of the *Alliaceae* and *Brassicaceae* families, whose lipid‐lowering effects have been largely proposed (Della Pepa et al. 2020; Feldman et al. 2021; Sun, Zhao, et al. 2021). One of the best characterized polyphenols from *Brassicaceae* plants is kaempferol (Cartea et al. 2010), which reduced TC levels in an animal model of overweight/obesity (Wang, Wu, and Zhao 2020). Quercetin, a polyphenol found in *Alliaceae* (El‐Saber Batiha et al. 2020; Kumar et al. 2022) and *Brassicaceae* (Cartea et al. 2010), has also shown lipid‐lowering effects in mice fed a high‐fat diet (Hoek‐van den Hil et al. 2014) and in apolipoprotein E (apoE) knockout mice (Jia et al. 2019). However, meta‐analyses of randomized controlled trials (RCTs) on the effects of quercetin supplementation on lipid profile in humans have produced conflicting results, from ineffectiveness (Sahebkar 2017) to significant reduction of TC and LDL‐C levels (Tabrizi et al. 2020). Therefore, the role of polyphenols in the beneficial effects of *Alliaceae* and *Brassicaceae* plants on lipid metabolism is far to be clarified, and other compounds could contribute to the health benefits of organosulfur‐containing plants. In this context, the potential hidden role of H_2_S released by polysulfides and isothiocyanates is a new and attractive hypothesis.

Clinical data on the efficacy of edible plants containing organosulfur compounds in patients with dyslipidemia are quite controversial. Previous systematic reviews have summarized the effects of garlic supplementation on serum lipids but have included both patients with dyslipidemia and healthy volunteers, thus analyzing a nonhomogeneous population. Furthermore, most of them have combined data from patients receiving only natural products with those from prevalent users of lipid‐lowering drugs (i.e., statins) (Fu et al. 2023; Khoo and Aziz 2009; Ried, Toben, and Fakler 2013; Stevinson, Pittler, and Ernst 2000), which are not directly comparable. This paper aims to summarize the results of controlled clinical trials on the potential lipid‐lowering properties of extracts containing organosulfur compounds in patients with dyslipidemia, to provide an updated state of the art and to open new perspectives in the nutraceutical/phytotherapeutic approach for the management of dyslipidemia.

## 2 Methods

The study was performed in compliance with the Preferred Reporting Items for Systematic reviews and Meta‐Analyses (PRISMA) guidelines. Protocol number: CRD42023446249 (registered in the PROSPERO database).

### 2.1 Study Selection

Medline, Embase, The Cochrane Central Register of Controlled Trials (CENTRAL), and SCOPUS databases were searched from inception to October 11, 2023. The search strategy was made of two parts: the first related to the treatment (i.e., edible plants of the *Alliaceae* or *Brassicaceae* families of traditional use), and the second related to the disease of interest (hypercholesterolemia or hypertriglyceridemia). The Boolean operator “AND” was used to combine the two parts (Data S1).

After the removal of duplicates and screening of titles/abstracts, the full texts of potentially eligible articles were retrieved, if available, and considered for inclusion. Two authors (E.P. and M.A.M.) carried out the screening process independently and discussed any disagreements with a third author (E.L.), who further checked the titles, abstracts, and full‐texts to clarify definitely whether or not the “controversial” records met the inclusion criteria. The web‐based screening tool for systematic reviews RAYYAN was used to manage the selection process (Ouzzani et al. 2016).

### 2.2 Eligibility Criteria

Inclusion criteria were listed according to the PICOD scheme. P (population): adult patients (≥ 18 years) with dyslipidemia (i.e., hypercholesterolemia, hypertriglyceridemia, or hyperlipidemia), regardless of gender or comorbidities. We accepted the definition of hypercholesterolemia, hypertriglyceridemia, or hyperlipidemia as reported in the original study. I (intervention): *Alliaceae* or *Brassicaceae* extracts, regardless of dosage or formulation. C (control): placebo or no treatment. O (outcome): change from baseline in lipid parameters reported in the studies, measured in serum or plasma. Safety and tolerability were secondary outcomes. D (study design): parallel and crossover controlled clinical studies, nonrandomized and randomized. Abstracts, reviews, study protocols, or studies published in languages other than English were excluded.

### 2.3 Data Extraction

For each record, two authors individually extracted the following information: (i) study design; (ii) inclusion criteria; (iii) number of patients, age, and gender; (iv) type of intervention, including botanical family, dosage, form of administration, and duration of treatment; (v) primary outcome (serum or plasma concentration of lipid parameters at baseline and at the end of the study); (vi) secondary outcome (safety and tolerability, if reported); and (vii) methodological quality of the study.

### 2.4 Risk of Bias and Certainty of Evidence Assessment

A modified version of the Cochrane risk of bias tool 2 (RoB 2) was used to evaluate the methodological quality of RCTs and crossover trials (Sterne et al. 2019). The tool for RCTs contains six domains, which consider potential deviations from declared interventions, randomization problems, unclear outcome measurement and reporting of results, and inadequate description of the administered products. The tool for crossover studies consists of an additional domain that refers to biases due to possible carryover effects. Domains are marked as “low risk,” “some concerns,” or as “high risk.” We assessed the risk of bias of nonrandomized controlled trials (NRCT) with the Cochrane ROBINS‐I tool, composed by seven domains (Sterne et al. 2016) that evaluate the risk of baseline confounding, selection process, and misclassification biases, as well as biases due to deviations from declared interventions, unclear outcome measurement and reporting of results, and inadequate description of the administered products. Domains are marked as “low risk,” “moderate risk,” “serious risk,” or “critical risk.” For each study, the global risk of bias was defined “high” when at least one domain was at “high risk” or “critical risk,” “moderate” when at least one domain was classified as “some concerns” or “moderate risk,” and “low” when all domains were at “low risk.”

The certainty (or quality) of evidence was assessed by two authors, independently, using the Grading of Recommendations, Assessment and Evaluation (GRADE) approach. The GRADE system consists of eight domains: (i) risk of bias; (ii) consistency of results; (iii) directness; (iv) precision; (v) publication bias; (vi) magnitude of the effect; (vii) plausible confounding; and (viii) dose–response gradient. For each outcome, the overall certainty of the evidence was classified as “high,” “moderate,” “low,” or “very low.”

### 2.5 Statistical Analysis

For each paper, the mean difference (MD) and standard deviation (SD) of lipid parameters between end‐of‐treatment and baseline (i.e., end‐of‐treatment levels minus baseline levels) were reported. If MD and SD were reported in mmol/L, we converted them to mg/dL by multiplying the value in mmol/L by 38.67 for TC, HDL‐C, and LDL‐C and by 88.57 for TGs (Rugge et al. 2011). When MD was not reported, we considered end‐of‐treatment and baseline mean values, if available. When SD was not available, we imputed it using the formula described in the Cochrane Handbook for Systematic Reviews of Interventions (section 6.5.2) (Cochrane 2023), considering a correlation coefficient of 0.8. This coefficient was chosen following the guidelines of the Cochrane Handbook, section 6.5.2 (Cochrane 2023). Briefly, we imputed the correlation coefficient of four studies with similar characteristics to those included in our review, which showed the SD for change from baseline to post‐intervention (Brull et al. 2015; Lee et al. 2016; Ried, Travica, and Sali 2016; Sangouni et al. 2021). For each study, we calculated the correlation coefficient as the mean of the correlations in the treatment and control arms and estimated the overall correlation coefficient as the mean of the four correlations. The resulting value was 0.79 (data not shown), which was approximated to 0.8.

When results were expressed as mean ± standard error of the mean (SEM), SD was calculated by multiplying SEM by the square root of the number of patients in each group.

We used random‐effect models considering the inverse variance method for study‐specific weights and the restricted maximum‐likelihood (REML) value for estimating tau squared (*τ* ^2^). We reported the data as MD between groups and relative 95% confidence interval (CI). For all parameters, a negative MD value indicated a greater decrease in concentration from baseline levels in the treatment group than in the control group. MD was considered significant when the corresponding 95% CI did not include the value 0.

Higgins' *I* ^2^ statistic was used to quantify heterogeneity. Heterogeneity was considered low if *I* ^2^ was less than 25%, moderate for values between 25% and 50%, and high for values above 50%. Cochrane's Q was used to test heterogeneity. Stratified analyses by the risk of bias were also performed. To test whether variables such as patients' lipid levels at the beginning of the study or duration of treatment could be related to the magnitude of the observed MD in lipid parameters, we performed meta‐regression analyses. We assessed the presence of publication bias by funnel plot visualization and Egger's test when appropriate (at least 10 studies available). Influence analyses were also performed by removing each study one by one. When we observed a change in the direction of pooled estimates, the omitted study was classified as influential. All the analyses were performed with R software version 4.2.3.

### 2.6 Safety Assessment

For the safety assessment, we first listed the side effects reported by the included studies. Then, for each study, we calculated the percentage of patients who experienced side effects out of the total number of patients. We reported the range between the calculated minimum and maximum percentage.

## 3 Results

### 3.1 Systematic Review

**FIGURE 2:**
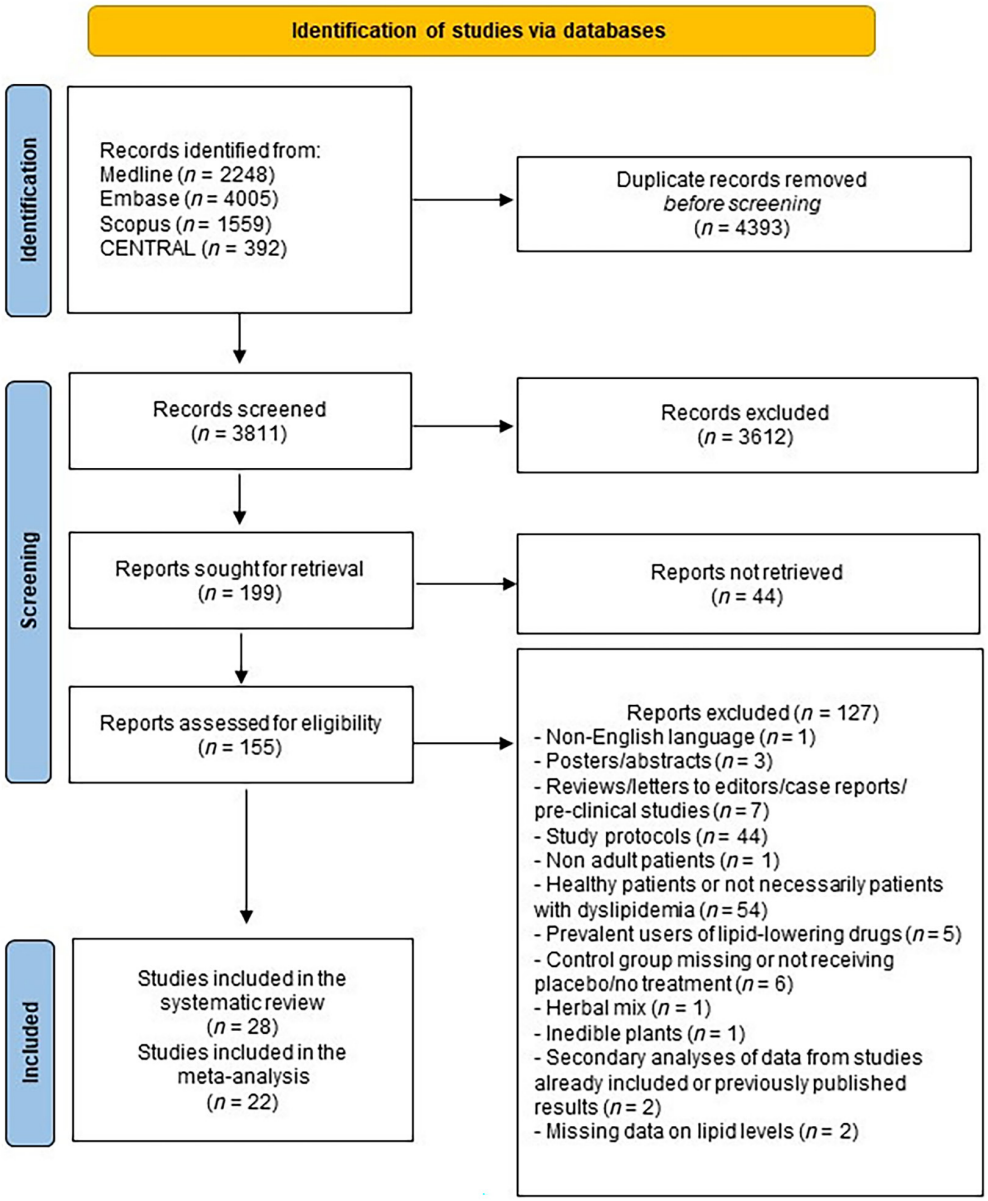
PRISMA flowchart.

We retrieved 2248 studies from Medline, 1559 from Scopus, 4005 from Embase, and 392 from CENTRAL searching (Figure 2). After the removal of duplicates, we screened 3811 records and assessed 155 studies for eligibility. Studies that met inclusion criteria (28) were included in the systematic review. The number of records that were excluded was 127, since they were: not written in English (1 record); poster/abstracts (3 records); reviews/letters to editors/case reports/preclinical studies (7 records); study protocols (44 records); studies on pediatric patients (1 record); studies on healthy patients or on patients not necessarily with dyslipidemia (i.e., dyslipidemia not considered among the inclusion criteria) (54 records); studies on prevalent users of lipid‐lowering drugs (5 records); without control group or with a control group not receiving placebo/no treatment (6 records); evaluating herbal mixtures (1 record); reporting previously published results or secondary analyses of data from studies already included (2 records); and with missing data on lipid levels (2 records).

Of the 28 studies, only one RCT (Aslani et al. 2016) and one of three study arms of another RCT (Gardner et al. 2007) assessed the efficacy of raw garlic, instead of plant extracts. Furthermore, only one article assessed the effectiveness of a combined treatment with garlic and statins in new users (Feng et al. 2022). Hence, they were excluded from the meta‐analysis. Three articles showed data in graphical form (Gillingham et al. 2011; Simons et al. 1995; Steiner et al. 1996), while one paper expressed the data in a way that was not relatable to what we decided to meta‐analyze (i.e., number of patients who achieved a marked change in blood lipid levels (Lau, Lam, and Wang‐Cheng 1987)). Therefore, 22 studies were included in the meta‐analysis.

Tables 1 and S1 show the results of the systematic review. Twenty‐two studies were RCTs, one was a NRCT, and five were randomized crossover trials. The mean age of patients was 52.2 ± 8.3 years in the control group and 51.7 ± 10.0 years in the intervention group, while 42.6% in the control group and 38.1% in the intervention group were men. Participants were subjects with: mild to moderate hypercholesterolemia, hypercholesterolemia (grade unknown), moderate hyperlipidemia, hyperlipidemia (grade unknown), chronic heart disease (CHD) and hypercholesterolemia, hypertriglyceridemia without severe dyslipidemia, coronary artery disease (CAD) and newly diagnosed dyslipidemia, kidney disease and dyslipidemia, renal transplant and hypercholesterolemia, or T2D and newly diagnosed dyslipidemia. For each study, the precise definition of dyslipidemia is given in Table S1.

**TABLE 1:** Summary of the included studies.

	No. of studies
Study design	
RCT (Adler and Holub 1997; Ashraf et al. 2005; Aslani et al. 2016; Bordia 1981; Feng et al. 2022; Gardner et al. 2007; Heshmat‐Ghahdarijani et al. 2022; Isaacsohn et al. 1998; Jain et al. 1993; Jia et al. 2020; Jung et al. 2014; Kannar et al. 2001; Kojuri, Vosoughi, and Akrami 2007; Lash et al. 1998; Lau, Lam, and Wang‐Cheng 1987; Lu et al. 2015; Neil et al. 1996; Peleg et al. 2003; Satitvipawee et al. 2003; Sobenin et al. 2008; Sobenin et al. 2010; Superko and Krauss 2000)	22
NRCT (Fatima et al. 2014)	1
Randomized crossover trial (Berthold, Sudhop, and von Bergmann 1998; Gillingham et al. 2011; Simons et al. 1995; Steiner et al. 1996; Valls et al. 2022)	5
Included patients^a^ (patients with)	
Mild‐to‐moderate hypercholesterolemia (Adler and Holub 1997; Aslani et al. 2016; Berthold, Sudhop, and von Bergmann 1998; Gardner et al. 2007; Jung et al. 2014; Lu et al. 2015; Peleg et al. 2003; Simons et al. 1995; Sobenin et al. 2008; Steiner et al. 1996; Superko and Krauss 2000; Valls et al. 2022)	12
Hypercholesterolemia (grade unknown) (Gillingham et al. 2011; Isaacsohn et al. 1998; Jain et al. 1993; Kannar et al. 2001; Satitvipawee et al. 2003)	5
Hyperlipidemia (grade unknown) (Fatima et al. 2014; Heshmat‐Ghahdarijani et al. 2022; Lau, Lam, and Wang‐Cheng 1987)	3
Chronic heart disease and hypercholesterolemia (Bordia 1981; Sobenin et al. 2010)	2
Moderate hyperlipidemia (Neil et al. 1996)	1
Others^b^	5
Type of intervention^c^	
*Alliaceae*	27
Garlic (Adler and Holub 1997; Ashraf et al. 2005; Aslani et al. 2016; Berthold, Sudhop, and von Bergmann 1998; Bordia 1981; Fatima et al. 2014; Feng et al. 2022; Gardner et al. 2007; Isaacsohn et al. 1998; Jain et al. 1993; Jia et al. 2020; Jung et al. 2014; Kannar et al. 2001; Kojuri, Vosoughi, and Akrami 2007; Lash et al. 1998; Lau, Lam, and Wang‐Cheng 1987; Neil et al. 1996; Peleg et al. 2003; Satitvipawee et al. 2003; Simons et al. 1995; Sobenin et al. 2008; Sobenin et al. 2010; Steiner et al. 1996; Superko and Krauss 2000; Valls et al. 2022)	25
Onion (Heshmat‐Ghahdarijani et al. 2022; Lu et al. 2015)	2
*Brassicaceae* (Canola oil) (Gillingham et al. 2011)	1
Measured parameters	
TC (Adler and Holub 1997; Ashraf et al. 2005; Aslani et al. 2016; Berthold, Sudhop, and von Bergmann 1998; Bordia 1981; Fatima et al. 2014; Feng et al. 2022; Gillingham et al. 2011; Heshmat‐Ghahdarijani et al. 2022; Isaacsohn et al. 1998; Jain et al. 1993; Jia et al. 2020; Jung et al. 2014; Kannar et al. 2001; Kojuri, Vosoughi, and Akrami 2007; Lash et al. 1998; Lau, Lam, and Wang‐Cheng 1987; Lu et al. 2015; Neil et al. 1996; Peleg et al. 2003; Satitvipawee et al. 2003; Simons et al. 1995; Sobenin et al. 2008; Sobenin et al. 2010; Steiner et al. 1996; Superko and Krauss 2000; Valls et al. 2022)	27
HDL‐C (Adler and Holub 1997; Ashraf et al. 2005; Aslani et al. 2016; Berthold, Sudhop, and von Bergmann 1998; Bordia 1981; Feng et al. 2022; Gardner et al. 2007; Gillingham et al. 2011; Heshmat‐Ghahdarijani et al. 2022; Isaacsohn et al. 1998; Jain et al. 1993; Jia et al. 2020; Jung et al. 2014; Kannar et al. 2001; Kojuri, Vosoughi, and Akrami 2007; Lash et al. 1998; Lu et al. 2015; Neil et al. 1996; Peleg et al. 2003; Satitvipawee et al. 2003; Simons et al. 1995; Sobenin et al. 2008; Sobenin et al. 2010; Steiner et al. 1996; Superko and Krauss 2000; Valls et al. 2022)	27
TGs (Adler and Holub 1997; Ashraf et al. 2005; Aslani et al. 2016; Berthold, Sudhop, and von Bergmann 1998; Fatima et al. 2014; Feng et al. 2022; Gardner et al. 2007; Gillingham et al. 2011; Heshmat‐Ghahdarijani et al. 2022; Isaacsohn et al. 1998; Jain et al. 1993; Jia et al. 2020; Jung et al. 2014; Kannar et al. 2001; Kojuri, Vosoughi, and Akrami 2007; Lash et al. 1998; Lau, Lam, and Wang‐Cheng 1987; Lu et al. 2015; Neil et al. 1996; Peleg et al. 2003; Satitvipawee et al. 2003; Simons et al. 1995; Sobenin et al. 2008; Sobenin et al. 2010; Steiner et al. 1996; Superko and Krauss 2000; Valls et al. 2022)	27
LDL‐C (Adler and Holub 1997; Ashraf et al. 2005; Aslani et al. 2016; Berthold, Sudhop, and von Bergmann 1998; Fatima et al. 2014; Feng et al. 2022; Gardner et al. 2007; Gillingham et al. 2011; Heshmat‐Ghahdarijani et al. 2022; Isaacsohn et al. 1998; Jain et al. 1993; Jia et al. 2020; Jung et al. 2014; Kannar et al. 2001; Kojuri, Vosoughi, and Akrami 2007; Lash et al. 1998; Lu et al. 2015; Neil et al. 1996; Peleg et al. 2003; Satitvipawee et al. 2003; Simons et al. 1995; Sobenin et al. 2008; Sobenin et al. 2010; Steiner et al. 1996; Superko and Krauss 2000; Valls et al. 2022)	26
Apo B (Isaacsohn et al. 1998; Jung et al. 2014; Neil et al. 1996; Superko and Krauss 2000; Valls et al. 2022)	5
Apo A1 (Isaacsohn et al. 1998; Jung et al. 2014; Neil et al. 1996; Valls et al. 2022)	4
LDL‐C/HDL‐C ratio (Gillingham et al. 2011; Kannar et al. 2001; Lu et al. 2015; Valls et al. 2022)	4
TC/HDL‐C ratio (Gardner et al. 2007; Gillingham et al. 2011; Valls et al. 2022)	3
Lp(a) (Isaacsohn et al. 1998; Simons et al. 1995; Superko and Krauss 2000)	3
Others^d^	8

Abbreviations: Apo, apolipoprotein; HDL‐C, high‐density lipoprotein cholesterol; LDL‐C, low‐density lipoprotein cholesterol; Lp(a), lipoprotein a; TC, total cholesterol; TGs, triglycerides.

^a^ Mean age (years): 52.2 (control group); 51.7 (intervention group). Men (%): 42.6 (control group); 38.1 (intervention group).

^b^ Hypertriglyceridemia without severe dyslipidemia (Jia et al. 2020); CAD and newly diagnosed dyslipidemia (Kojuri, Vosoughi, and Akrami 2007); kidney disease and dyslipidemia (Feng et al. 2022); renal transplant patients with hypercholesterolemia (Lash et al. 1998); and type 2 diabetes and newly diagnosed dyslipidemia (Ashraf et al. 2005).

^c^ Length of treatment: 26 days–12 months. Daily dosage: 4–20 g for raw garlic; 0.25–7.2 g for dried extracts; unknown for Canola oil.

^d^ LDL‐C + very low‐density lipoprotein cholesterol (VLDL‐C), phospholipids (PLs), and TC/PLs ratio (Bordia 1981); non‐HDL‐C (Gillingham et al. 2011); Apo B/Apo A1 ratio (Valls et al. 2022); TGs/Apo B (Valls et al. 2022); free fatty acids (FFAs) (Jung et al. 2014); lathosterol/TC ratio, LDL receptor mass, and LDL oxidation rate (Simons et al. 1995); LDL diameter (Superko and Krauss 2000); and nonesterified fatty acids (NEFAs) (Valls et al. 2022).

Almost all of the included studies evaluated the efficacy of *Alliaceae* extracts: 25 garlic and 2 onions. Only one study assessed the effects of canola oil (*Brassica napus* L., *Brassicaceae*) (Gillingham et al. 2011), thus limiting the generalizability of our results to all edible plants containing organosulfur compounds. The daily dosage was 4–20 g for raw garlic (Aslani et al. 2016; Gardner et al. 2007) and 0.25–7.2 g for dried extracts (Berthold, Sudhop, and von Bergmann 1998; Steiner et al. 1996), while the treatment duration ranged from 26 days (Gillingham et al. 2011) to 10 months (Bordia 1981), although half of the records selected a 12‐week period (Adler and Holub 1997; Ashraf et al. 2005; Berthold, Sudhop, and von Bergmann 1998; Fatima et al. 2014; Feng et al. 2022; Isaacsohn et al. 1998; Jain et al. 1993; Jia et al. 2020; Jung et al. 2014; Kannar et al. 2001; Lash et al. 1998; Satitvipawee et al. 2003; Simons et al. 1995; Sobenin et al. 2008; Superko and Krauss 2000).

Most articles measured TC (except for (Gardner et al. 2007)), HDL‐C (except for (Lau, Lam, and Wang‐Cheng 1987)), and TGs (except for (Bordia 1981)) at the end of treatment. Out of 28 studies, 26 studies also reported LDL‐C levels (Adler and Holub 1997; Ashraf et al. 2005; Aslani et al. 2016; Berthold, Sudhop, and von Bergmann 1998; Fatima et al. 2014; Feng et al. 2022; Gardner et al. 2007; Gillingham et al. 2011; Heshmat‐Ghahdarijani et al. 2022; Isaacsohn et al. 1998; Jain et al. 1993; Jia et al. 2020; Jung et al. 2014; Kannar et al. 2001; Kojuri, Vosoughi, and Akrami 2007; Lash et al. 1998; Lu et al. 2015; Neil et al. 1996; Peleg et al. 2003; Satitvipawee et al. 2003; Simons et al. 1995; Sobenin et al. 2008; Steiner et al. 1996; Superko and Krauss 2000; Valls et al. 2022). Other parameters were: LDL‐C + very low‐density lipoprotein cholesterol (VLDL‐C), phospholipids (PLs) and TC/PLs ratio (Bordia 1981); TC/HDL‐C ratio (Gardner et al. 2007; Gillingham et al. 2011; Valls et al. 2022); LDL‐C/HDL‐C ratio (Gillingham et al. 2011; Kannar et al. 2001; Lu et al. 2015; Valls et al. 2022); non‐HDL‐C (Gillingham et al. 2011); apolipoprotein (Apo) A1 (Isaacsohn et al. 1998; Jung et al. 2014; Neil et al. 1996; Valls et al. 2022) and Apo B (Isaacsohn et al. 1998; Jung et al. 2014; Neil et al. 1996; Superko and Krauss 2000; Valls et al. 2022); Apo B/Apo A1 ratio (Valls et al. 2022); TGs/Apo B (Valls et al. 2022); lipoprotein a (Lp(a)) (Isaacsohn et al. 1998; Simons et al. 1995; Superko and Krauss 2000); free fatty acids (FFAs (Jung et al. 2014)); lathosterol/TC ratio, LDL receptor mass, LDL oxidation rate (Simons et al. 1995); LDL diameter (Superko and Krauss 2000); and nonesterified fatty acids (NEFAs) (Valls et al. 2022).

### 3.2 Risk of Bias

**FIGURE 3:**
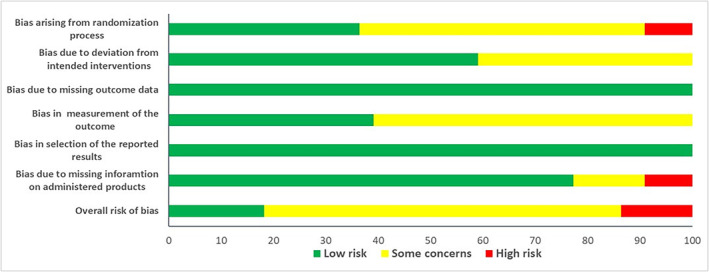
Risk of bias of randomized controlled studies (RCTs).

Three RCTs were at “high” risk of bias (Bordia 1981; Lau, Lam, and Wang‐Cheng 1987; Lu et al. 2015), 15 at “moderate” risk of bias (Adler and Holub 1997; Ashraf et al. 2005; Aslani et al. 2016; Feng et al. 2022; Gardner et al. 2007; Heshmat‐Ghahdarijani et al. 2022; Isaacsohn et al. 1998; Jain et al. 1993; Jia et al. 2020; Jung et al. 2014; Kannar et al. 2001; Kojuri, Vosoughi, and Akrami 2007; Lash et al. 1998; Peleg et al. 2003; Sobenin et al. 2010), and 4 at “low” risk of bias (Neil et al. 1996; Satitvipawee et al. 2003; Sobenin et al. 2008; Superko and Krauss 2000) (Figures 3 and S1). In general, the main critical issues concerned the randomization and blinding processes, as well as the insufficient or missing description of the standardization/composition of the administered products.

NRCT (Fatima et al. 2014) was labeled as a study with “low” methodological quality (data not shown), due to potential biases in the classification of the interventions and outcome measurement. The lack of information on the administered product further contributed to the low methodological quality of the study.

**FIGURE 4:**
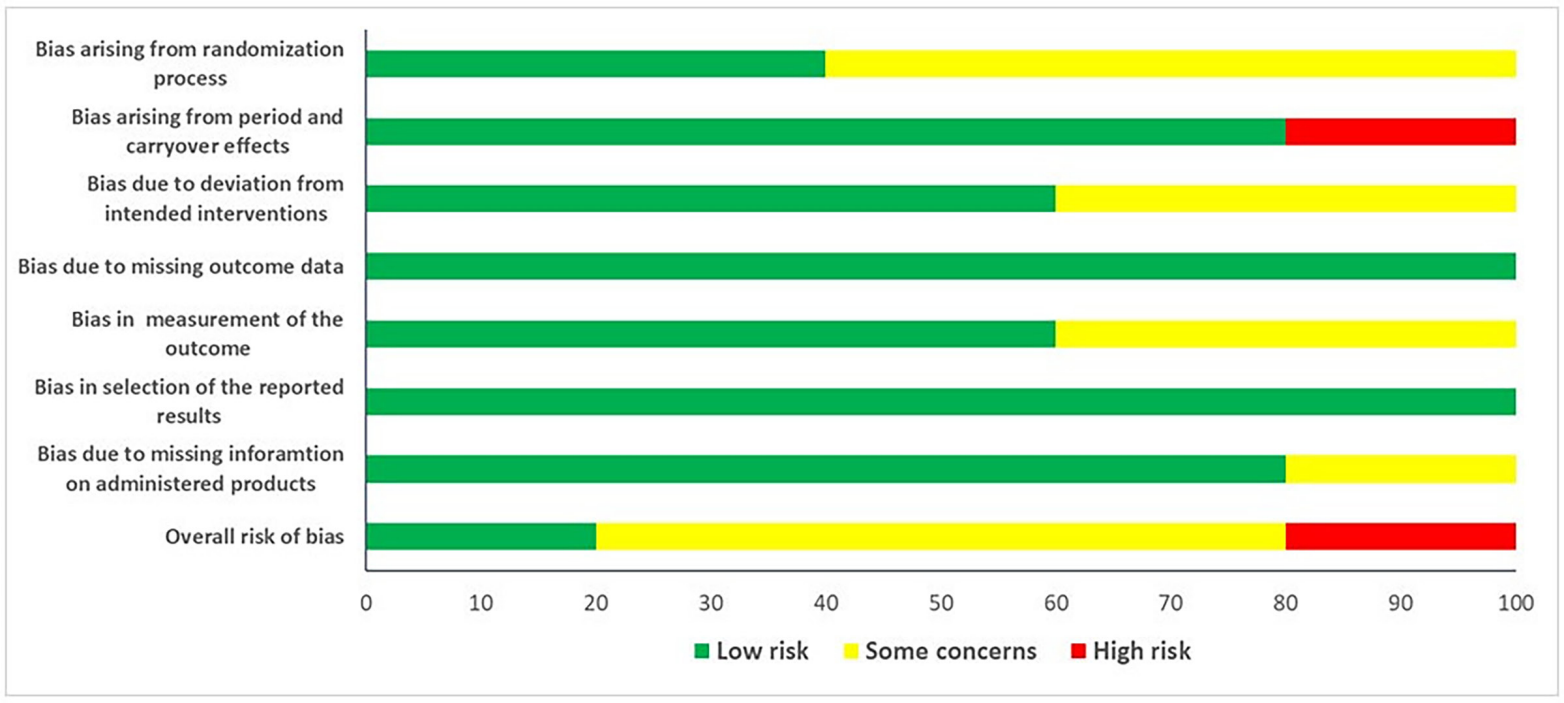
Risk of bias of crossover trials.

Among randomized crossover trials, one was classified at “high” risk of bias (Steiner et al. 1996), three at “moderate” risk of bias (Berthold, Sudhop, and von Bergmann 1998; Gillingham et al. 2011; Simons et al. 1995), and one at “low” risk of bias (Valls et al. 2022) (Figures 4 and S2). The “high” risk of bias of the study by Steiner and colleagues (Steiner et al. 1996) was due to potential carryover effects, while the studies with “moderate” methodological quality showed some critical issues in the randomization and blinding processes.

### 3.3 Results of Meta‐Analysis

#### 3.3.1 Effects of *Alliaceae* Extracts on TC and LDL‐C

**FIGURE 5:**
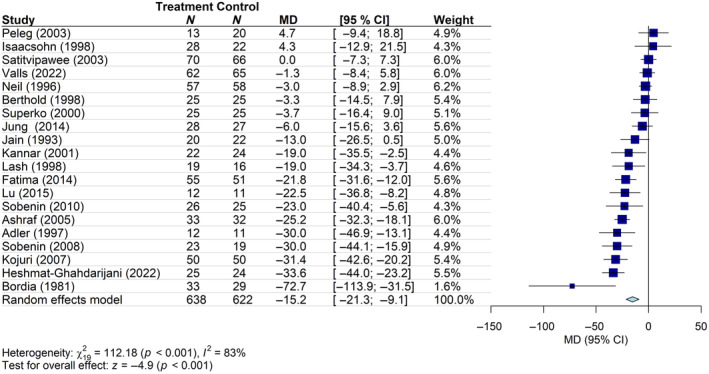
Forest plot of the mean difference (MD) of TC levels (mg/dL) with 95% confidence interval (CI) in patients receiving *Alliaceae* extracts (treatment group) versus placebo/no treatment (control group). Garlic: 18 studies; onion: 2 studies (Heshmat‐Ghahdarijani et al. 2022; Lu et al. 2015).

The meta‐analysis of 20 studies (622 patients in the control group and 638 in the intervention group) demonstrated that the daily use of *Alliaceae* extracts (garlic: 18 studies; onion: 2 studies) leads to a significant reduction in TC levels (mean change in TC from the baseline vs. placebo/no treatment: −15.2 mg/dL; 95% CI: −21.3, −9.1), with a high heterogeneity (*I* ^2^ = 83%) (Figure 5). Significant differences between subgroups were found by stratifying results by the risk of bias (*p* = 0.02) (Figure S3). In particular, the reduction in TC levels resulted from studies with “high” and “moderate” risks of bias and not from those with “low” risk of bias, where the result was not statistically significant. Meta‐regression models analyzing the possible association between effect size and baseline TC levels or effect size and duration of the intervention were not significant. Influence analyses demonstrated no change in the direction of the effect (data not shown). Examination of the funnel plots showed small asymmetries, but Egger's test results did not suggest the presence of significant publication bias in the meta‐analysis (*p* = 0.08) (Figure S4).

The overall certainty of evidence for this outcome, assessed using the GRADE approach, was rated as “low” (Table S2), mainly due to the risk of bias and high heterogeneity (inconsistency) of the included studies. Therefore, the “real” effect may be substantially different from the estimated effect.

**FIGURE 6:**
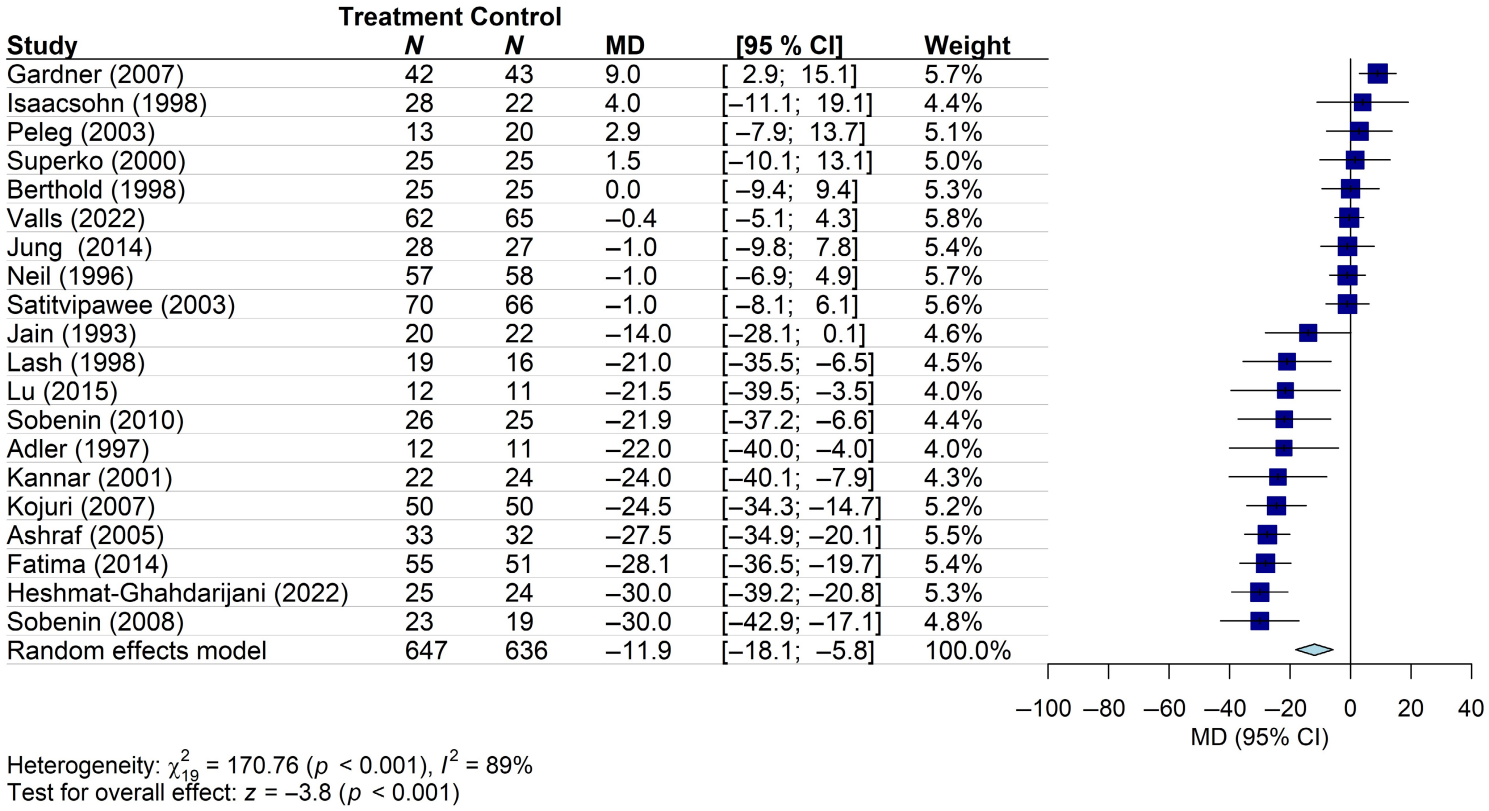
Forest plot of the mean difference (MD) of LDL‐C levels (mg/dL) with 95% confidence interval (CI) in patients receiving *Alliaceae* extracts (treatment group) versus placebo/no treatment (control group). Garlic: 18 studies; onion: 2 studies (Heshmat‐Ghahdarijani et al. 2022; Lu et al. 2015).

Treatment with *Alliaceae* extracts (garlic: 18 studies; onion: 2 studies) also reduced LDL‐C levels in patients with hypercholesterolemia (636 patients in the control group and 647 in the intervention group) (mean change in LDL‐C from the baseline: −11.9 mg/dL; 95% CI: −18.1, −5.8), with a high heterogeneity (*I* ^2^ = 89%) (Figure 6). In the analysis stratified by risk of bias, differences emerged between the subgroups (*p* = 0.002). In particular, the significant reduction in LDL‐C levels observed in patients belonging to the intervention group resulted from studies characterized by “high” risk of bias and not from those with “moderate” or “low” risk of bias, where the result was not statistically significant (Figure S5). Meta‐regression models analyzing the possible association between the effect size and baseline LDL‐C levels, as well as the duration of intervention, were not significant (data not shown). No change in the direction of pooled estimates was observed in the influence analysis (data not shown). Examination of the funnel plots showed small asymmetries, but Egger's test results did not suggest the presence of significant publication bias in the meta‐analysis (*p* = 0.05) (Figure S6).

The overall certainty of evidence for this outcome, assessed using the GRADE approach, was rated as “low” (Table S2), mainly due to the risk of bias and high heterogeneity of the included studies.

Studies excluded from the meta‐analysis (i.e., those evaluating the effectiveness of raw garlic instead of plant extracts or a combined treatment with garlic and statins in new users, those showing results in the graphical form, or those expressing the data as number of patients who achieved a marked change in blood lipid levels) showed a similar scenario. Daily consumption of raw garlic bulb for 8 weeks, as well as liquid garlic bulb extract and aged garlic bulb extract (AGE) for 24 weeks, led to a marked reduction in TC (Aslani et al. 2016; Lau, Lam, and Wang‐Cheng 1987; Steiner et al. 1996) and LDL‐C levels (Aslani et al. 2016; Steiner et al. 1996) in patients with dyslipidemia. In contrast, one article showed no significant change in lipid parameters in subjects receiving dried garlic bulb extract (Simons et al. 1995) for 12 weeks compared to the placebo group. Only one study evaluated the efficacy of a combined treatment with garlic extract and atorvastatin in new users (Feng et al. 2022) and demonstrated a more pronounced reduction in TC and LDL‐C levels in patients receiving garlic plus statin versus patients receiving only the “traditional” lipid‐lowering drug. Finally, the study on patients consuming a diet rich in canola oil (*B. napus* L., *Brassicaceae*) for 26 days showed a marked reduction in both TC and LDL‐C levels compared with the control group (Gillingham et al. 2011).

#### 3.3.2 Effects of *Alliaceae* Extracts on HDL‐C and TGs

**FIGURE 7:**
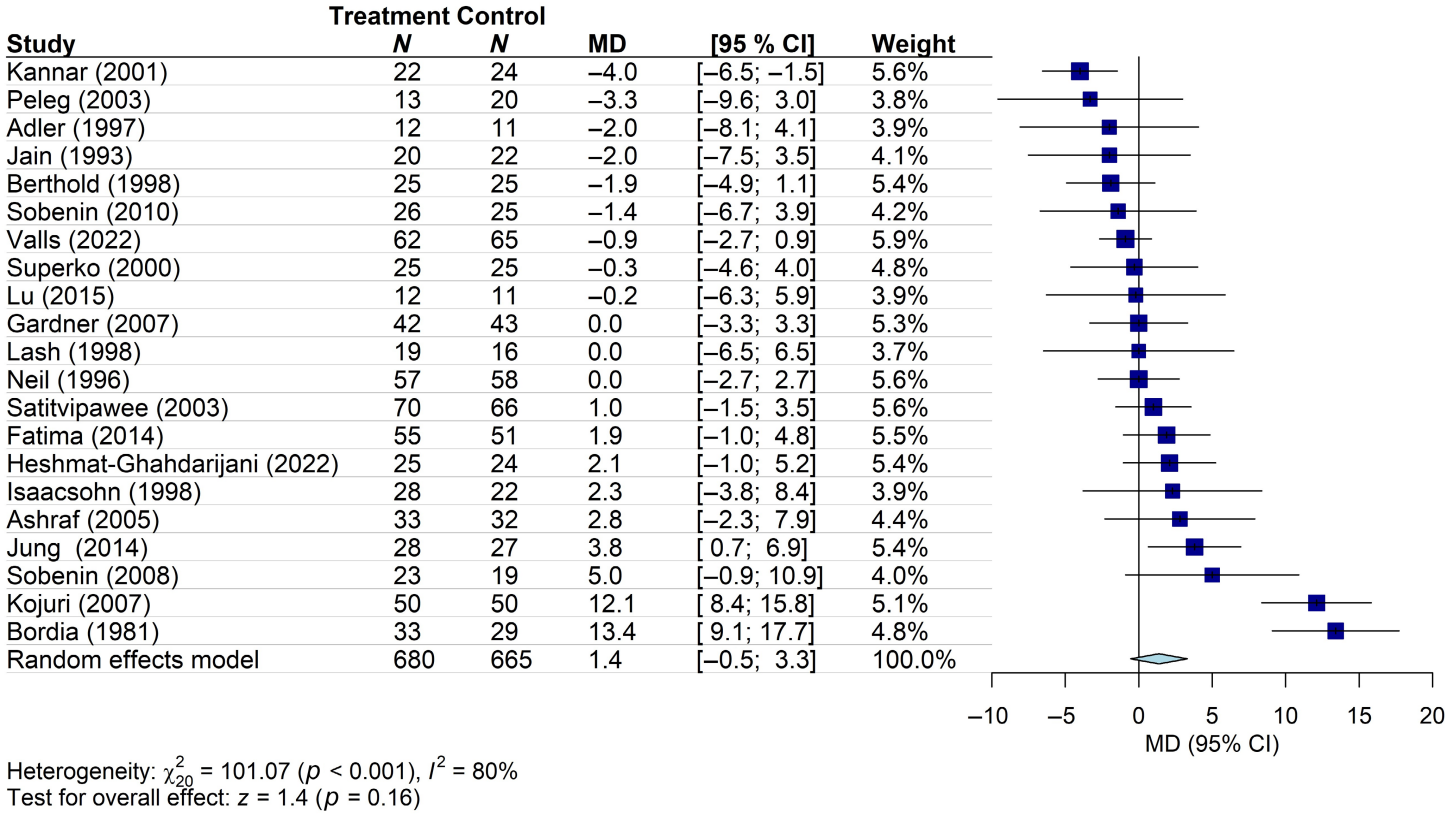
Forest plot of the mean difference (MD) of HDL‐C levels (mg/dl) with 95% confidence interval (CI) in patients receiving *Alliaceae* extracts (treatment group) versus placebo/no treatment (control group). Garlic: 19 studies; onion: 2 studies (Heshmat‐Ghahdarijani et al. 2022; Lu et al. 2015).

The meta‐analysis of 21 studies (665 patients in the control group and 680 in the intervention group) demonstrated that *Alliaceae* extracts (garlic: 19 studies; onion: 2 studies) produced a slight increase in HDL‐C levels versus placebo/no treatment in patients with dyslipidemia. However, the result was not statistically significant (mean change in HDL‐C from the baseline: 1.4 mg/dL; 95% CI: −0.5, 3.3), and the meta‐analysis showed a high heterogeneity (*I* ^2^ = 80%) (Figure 7). There was no significant difference between subgroups when results were stratified by the risk of bias (*p* = 0.43) (Figure S7). In the meta‐regression analysis, the association between HDL‐C levels at the baseline, or the duration of the intervention, and the change in HDL‐C levels at the end of the treatment was not significant (data not shown). Influence analyses showed no significant effects (data not shown), and no evidence of publication bias was observed (*p* = 0.33) (Figure S8).

The overall certainty of evidence for this outcome, assessed using the GRADE approach, was rated as “low” (Table S2), mainly due to high heterogeneity and imprecision of the included studies.

Only three studies (154 patients in the control group and 229 in the intervention group) reported the effects of garlic extracts on TG levels in patients with hypertriglyceridemia, while none examined those of onion. The results showed that daily treatment with garlic extracts, compared with placebo/no treatment, reduces TGs (mean change in TGs from the baseline: −16.7 mg/dL; 95% CI: −52.0, 18.7), with a high heterogeneity (*I* ^2^ = 88%) (Figure S9). This reduction, which was not statistically significant, is the result of the single study conducted in patients with higher TG levels at the baseline (more than 300 mg/dL) (Jia et al. 2020). In the influence analysis, removing this study changed the direction of the overall effect (increase in TG levels) but still not to a significant level. An asymmetry in the distribution of studies seemed to emerge from the TG funnel plot (Figure S10).

The overall certainty of evidence for this outcome, assessed using the GRADE approach, was rated as “very low” (Table S2), mainly due to the risk of bias, indirectness, imprecision, and high heterogeneity (inconsistency) of the included studies. Therefore, the “real” effect is likely to be substantially different from the estimated effect.

Articles not included in the meta‐analysis (as reported in Section 3.3.1) demonstrated that the daily consumption of raw garlic bulb for 8 weeks and AGE for 24 weeks led to a slight increase in HDL‐C levels (Aslani et al. 2016) and ratio between HDL‐C and TC (Steiner et al. 1996), respectively. In contrast, one article did not show significant changes in HDL‐C levels in patients receiving dried garlic bulb extract (Simons et al. 1995) for 12 weeks versus the placebo group. The only one article evaluating the efficacy of a combined treatment with garlic bulb extract and atorvastatin (Feng et al. 2022) demonstrated a more pronounced change in HDL‐C (increase) and TGs (decrease) in patients receiving garlic plus statin compared to patients treated with the “traditional” lipid‐lowering drug. Also, consumption of liquid garlic bulb extract significantly reduced TG levels in patients with dyslipidemia after 24 weeks (Lau, Lam, and Wang‐Cheng 1987). Finally, the study on patients who consumed a diet rich in canola oil (*B. napus* L., *Brassicaceae*) for 26 days showed no change in HDL‐C levels (Gillingham et al. 2011).

#### 3.3.3 Safety and Tolerability

Fifteen studies provided data on the safety of garlic extracts in patients with dyslipidemia. The most reported side effects were gastrointestinal disorders, including bad body and breath odor (Gardner et al. 2007; Isaacsohn et al. 1998; Jain et al. 1993; Kannar et al. 2001; Neil et al. 1996; Simons et al. 1995), flatulence/nausea (Gardner et al. 2007; Lau, Lam, and Wang‐Cheng 1987; Simons et al. 1995), diarrhea (Bordia 1981; Lash et al. 1998), heartburn (Ashraf et al. 2005), abdominal discomfort (Isaacsohn et al. 1998), dyspepsia (Jia et al. 2020), and epigastric pain (Lash et al. 1998). In some cases, these side effects have led to treatment discontinuation (Table 2).

**TABLE 2:** Most common side effects associated with the use of *Alliaceae* extracts.

Side effect	No. of studies	Proportion (range)	Discontinuations due to side effects
Bad body and breath odor	6	0.05–0.90	Yes
Flatulence/nausea	3	0.04–0.11	Yes
Diarrhea	2	0.03–0.05	Yes
Heartburn	1	0.03	Yes
Abdominal discomfort	1	0.07	Yes
Dyspepsia	1	0.007	Yes
Epigastric pain	1	0.05	No

## 4 Discussion

**DIAGRAM 1:**
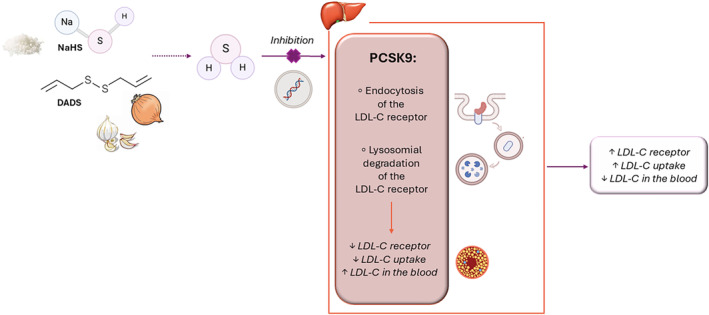
Proposed mechanism for the lipid‐lowering effects of natural and synthetic H_2_S‐donors. Proprotein convertase subtilisin/kexin type 9 (PCSK9) induces degradation of the low‐density lipoprotein cholesterol (LDL‐C) receptor, which is expressed on the cell membrane of hepatocytes and contributes to the uptake of LDL‐C from the bloodstream. Briefly, PCSK9 promotes LDL‐C receptor endocytosis and the subsequent degradation in lysosomes. This event leads to a reduction in LDL‐C receptor expression and, therefore, LDL‐C uptake by hepatocytes, thereby increasing LDL‐C levels in the blood. The H_2_S‐donors sodium hydrosulfide (NaHS) and diallyl disulfide (DADS) reduce PCSK9 expression and, thus, preserve LDL‐C receptor density on the cell membrane and increase LDL‐C uptake by hepatocytes.

The progressive increase in the global prevalence of dyslipidemia represents a serious threat to healthcare systems. Indeed, the imbalance in blood lipid levels can gradually increase the risk of hospitalization and death from CVD over time (Du and Qin 2023), even in the absence of clinically obvious signs and symptoms. Therefore, dyslipidemia can be considered one of the major “silent killers” of the 21st century (Tan et al. 2018). The identification of new therapeutic options (e.g., lipid‐lowering drugs, foods, or extracts) is an urgent need to improve the global health and financial burden of dyslipidemia. In this scenario, the use of natural products is considered a strategy of great pharmacological and nutraceutical interest. Among these, edible plants containing organosulfur compounds are emerging as potential regulators of lipid metabolism, partly due to the ability of *Alliaceae* polysulfides and *Brassicaceae* isothiocyanates to release the gasotransmitter H_2_S. An intriguing explanation for the possible role of H_2_S in the lipid‐lowering effects of natural sulfur compounds was provided by two independent preclinical studies, which demonstrated that the H_2_S‐donors NaHS and diallyl disulfide (DADS) reduce the expression of proprotein convertase subtilisin/kexin type 9 (PCSK9), thus increasing the uptake of LDL‐C by hepatocytes (Wu et al. 2021; Xiao et al. 2019) (Diagram 1).

Given the growing interest in natural H_2_S‐donors, we have summarized the main findings of clinical studies on the potential efficacy of extracts containing organosulfur compounds in patients with dyslipidemia. However, we realized that the number of studies evaluating the lipid‐lowering effects of *Brassicaceae* extracts is very low and does not allow us to generalize our results to all edible plants rich in organosulfur compounds. Only one of the included clinical trials investigated the effects of canola oil (*B. napus* L., *Brassicaceae*) and showed a marked reduction in both TC and LDL‐C levels at the end of the treatment (Gillingham et al. 2011).

All the other studies evaluated the potential efficacy of garlic extracts and, very few, onion extracts. In this review, we have demonstrated that the use of garlic and onion extracts leads to a significant reduction in TC and LDL‐C levels in hypercholesterolemic patients. Heterogeneity was consistent, but when we performed meta‐regression analyses, we found no significant association between TC and LDL‐C levels at the baseline, as well as the duration of treatment, and the effects of the intervention. We are aware that studies at “moderate” to “high” methodological quality may have influenced the results of our meta‐analysis and that the certainty of the evidence was “low” to “very low” for all outcomes. These are limitations common to all meta‐analyses that summarize the results on the efficacy of natural products, since the included trials usually have variability in study protocols, selection of interventions, and participants. To partially overcome the last criticality, we deliberately excluded studies on healthy volunteers or prevalent users of lipid‐lowering drugs to provide results on a homogeneous population. Indeed, the comparison of such different populations, carried out in previous meta‐analyses (Fu et al. 2023; Khoo and Aziz 2009; Ried, Toben, and Fakler 2013; Stevinson, Pittler, and Ernst 2000), could lead to wrong conclusions.

In this paper, we also investigated the potential efficacy of garlic and onion extracts in increasing HDL‐C levels, but the results were not significant. We have not evaluated the effectiveness of garlic and onion on other parameters (e.g., TC/HDL‐c ratio and non‐HDL‐C) due to the low number of studies reporting these indicators. As known, both parameters are powerful predictors of CV outcomes in subjects with hypercholesterolemia (Calling et al. 2019; Calling et al. 2021) and can be easily measured/interpreted in the clinical practice to identify future patients with CVDs. Therefore, these parameters should be evaluated to confirm and strengthen our findings. Finally, we studied the potential efficacy of garlic extracts on TG levels in patients with hypertriglyceridemia, but the low number of studies does not allow us to provide definitive conclusions and make valid inferences. Future studies are needed to corroborate this promising result.

Safety results showed that garlic extracts are well‐tolerated, although gastrointestinal events often led to treatment discontinuation. The most frequent were mild side effects, such as bad body and breath odor, flatulence, and nausea. This evidence is consistent with previous studies (Piragine, Petri, et al. 2022), in which the glucose‐lowering properties of *Alliaceae* and *Brassicaceae* plants were demonstrated in patients with T2D without recording any toxicity. Of note, the effects on blood glucose levels promoted in healthy people were not clinically significant, suggesting that the use of organosulfur‐containing supplements can be considered safe and tolerable in the “general population.” In fact, onion peel extract did not promote hypoglycemic effects in overweight patients without diabetes (Lee et al. 2016), and garlic extract did not change blood glucose levels in subjects with prediabetes (Park et al. 2020). However, safety was not our primary outcome, and the exclusion of observational studies limited the possibility to identify rare adverse events in the “real‐world” population. Therefore, we cannot further speculate on the safety and tolerability of these natural products after prolonged use. In addition, no information on the effects of *Alliaceae* and *Brassicaceae* extracts, or their isolated compounds, on kidney and liver functions has emerged in the included studies. Preclinical studies have shown that garlic extracts significantly prevented renal and hepatic damage in the rat models of diabetes (Al‐Qattan et al. 2016; Saryono et al. 2022). The same effects have been demonstrated for isolated organosulfur compounds, such as allicin (Garcia‐Trejo et al. 2016; Garcia Trejo et al. 2017), DADS (Sharma et al. 2021), and the isothiocyanate sulforaphane (from *B. oleracea* L., broccoli) (Monteiro et al. 2023). Subchronic administration of the isothiocyanate erucin (from *E. sativa* Mill., rocket salad) produced a significant reduction of creatinine levels in healthy rats at the highest dose tested (40 mg/kg), thus indicating potential toxicity. However, it was well‐tolerated at lower doses (10 and 2.5 mg/kg), which are more compatible with dietary vegetable intake or regular use of organosulfur‐containing supplements (Kaur et al. 2022).

## 5 Conclusions and Future Directions

The results of this study demonstrate that *Alliaceae* extracts significantly reduce TC and LDL‐C levels in patients with hypercholesterolemia, supporting the use of garlic and onion extracts in a homogeneous and specific (i.e., “drug‐free”) patient cohort. At the same time, this systematic review highlights the lack of information on the potential lipid‐lowering properties of *Brassicaceae* extracts and strongly suggests the need for new studies on these apparently “forgotten” natural products, opening new perspectives in the clinical management of dyslipidemia. In our opinion, as observed for garlic and onion extracts, *Brassicaceae* supplements could also have high nutraceutical/phytotherapeutic potential due to the high amount of organosulfur compounds: they could slow down the progression of dyslipidemia and prevent the onset of CV complications. In this regard, the study of the potential association between the regular use of *Alliaceae* or *Brassicaceae* extracts and CV risk reduction in patients with hypercholesterolemia could be a future direction of our work. Finally, defining an optimal dose of natural products, providing better standardization in organosulfur compounds, and performing new clinical trials with high methodological quality represent urgent needs in this promising research field.

This systematic review with meta‐analysis has many strengths. First, it is the first study to summarize the effects of supplements containing organosulfur compounds in patients with dyslipidemia, as well as the first meta‐analysis on dyslipidemic patients rather than healthy volunteers. To the best of our knowledge, this is also the first systematic review on the topic in which the certainty of evidence of the included studies was assessed with the rigorous GRADE approach.

However, a limitation of this work is the deliberate exclusion of observational studies. This does not allow us to identify rare adverse events in the “real‐world” population, nor to demonstrate the efficacy of *Alliaceae* and *Brassicaceae* extracts after prolonged use. Therefore, future studies are needed to corroborate our results and confirm the safety and tolerability of organosulfur‐containing extracts (e.g., in terms of kidney and liver functions) in the “real‐world” setting.

## Author Contributions

**Eugenia Piragine:** conceptualization, data curation, methodology, writing – original draft, writing – review and editing. **Marco Andrea Malanima:** data curation, methodology, writing – original draft, writing – review and editing. **Costanza Ceccanti:** writing – review and editing. **Lucia Guidi:** funding acquisition, project administration, writing – review and editing. **Alma Martelli:** funding acquisition, project administration, supervision, writing – review and editing. **Ersilia Lucenteforte:** data curation, methodology, supervision, writing – review and editing. **Vincenzo Calderone:** conceptualization, supervision, writing – review and editing.

## Consent

The authors have nothing to report.

## Conflicts of Interest

The authors declare no conflicts of interest.

## Supporting information


Data S1.

Data S2.

## Data Availability

The data are available from the corresponding author upon request.
