# Supplementary material for: Alliaceae versus Brassicaceae for Dyslipidemia: State of the Art and Future Perspectives. Systematic Review and Meta‐Analysis of Clinical Studies
Source: Phytother Res. 2024 Sep 29;38(12):5765–81. doi: 10.1002/ptr.8350 (PMC11634823; doi:10.1002/ptr.8350)
Supplement: Supplementary file 1 — Data S1. [file PTR-38-5765-s001.docx]

**S1. Search strategy.**

**Medline**

(Alliaceae OR garlic OR (garlic AND extract*) OR Allium OR (Allium AND sativum) OR onion OR (onion AND extract*) OR (Allium AND cepa) OR Brassicaceae OR Crucifers OR broccoli OR (broccoli AND extract*) OR rocket OR (rocket AND salad) OR cauliflower OR (cauliflower AND extract*)) AND (hypercholesterolemia OR hypertriglyceridemia OR hyperlipidemia OR hyperlipoproteinemia OR hypocholesterolemic OR (metabolic AND syndrome) OR (metabolic AND disease*) OR cholesterol OR (Low-density AND lipoprotein*) OR lipoprotein* OR LDL OR HDL OR (High-density AND lipoprotein*) OR (total AND cholesterol) OR triglyceride* OR lipid OR (lipid AND metabolism) OR antihyperlipidemic) NOT (review OR case report* OR editorial OR meta-analysis). Filters: humans and English.

**Scopus**

TITLE-ABS-KEY ( alliaceae OR garlic OR ( garlic AND extract* ) OR allium OR ( allium AND sativum ) OR onion OR ( onion AND extract* ) OR ( allium AND cepa ) OR brassicaceae OR crucifers OR broccoli OR ( broccoli AND etract* ) OR rocket OR ( rocket AND salad ) OR cauliflower OR ( cauliflower AND extract*)) AND TITLE-ABS-KEY ( hypercholesterolemia OR hypertriglyceridemia OR hyperlipidemia OR hyperlipoproteinemia OR hypocholesterolemic OR ( metabolic AND syndrome ) OR ( metabolic AND disease* ) OR cholesterol OR ( low-density AND lipoprotein* ) OR lipoprotein* OR LDL OR HDL OR ( high-density AND lipoprotein* ) OR ( total AND cholesterol ) OR triglyceride* OR lipid OR ( lipid AND metabolism ) OR antihyperlipidemic ) AND NOT TITLE-ABS-KEY ( review OR case AND report* OR editorial OR meta-analysis ) AND ( LIMIT-TO ( LANGUAGE , "English" ) ) AND ( LIMIT-TO ( EXACTKEYWORD , "Human") OR LIMIT-TO ( EXACTKEYWORD , "Humans")).

**CENTRAL**

((Alliaceae OR garlic OR (garlic AND extract*) OR Allium OR (Allium AND sativum) OR onion OR (onion AND extract*) OR (Allium AND cepa) OR Brassicaceae OR Crucifers OR broccoli OR (broccoli AND extract*) OR rocket OR (rocket AND salad) OR cauliflower OR (cauliflower AND extract*))):ti,ab,kw AND ((hypercholesterolemia OR hypertriglyceridemia OR hyperlipidemia OR hyperlipoproteinemia OR hypocholesterolemic OR (metabolic AND syndrome) OR (metabolic AND disease*) OR cholesterol OR (low-density AND lipoprotein*) OR lipoprotein* OR LDL OR HDL OR (high-density AND lipoprotein*) OR (total AND cholesterol) OR triglyceride* OR lipid OR (lipid AND metabolism) OR antihyperlipidemic)):ti,ab,kw NOT ((review OR case report* OR editorial OR meta-analysis)):ti,ab,kw. Filter: English.

**Embase**

#6. #1 AND #2 NOT #3 AND [humans]/lim AND [english]/lim

#5. #1 AND #2 NOT #3

#4. #1 AND #2

#3. ('review'/exp OR review OR case) AND report* OR 'editorial'/exp OR editorial OR 'meta analysis'/exp OR 'meta analysis'

#2. 'hypercholesterolemia'/exp OR hypercholesterolemia OR 'hypertriglyceridemia'/exp OR hypertriglyceridemia OR 'hyperlipidemia'/exp OR hyperlipidemia OR 'hyperlipoproteinemia'/exp OR hyperlipoproteinemia OR hypocholesterolemic OR (metabolic AND ('syndrome'/exp OR syndrome)) OR (metabolic AND disease*) OR 'cholesterol'/exp OR cholesterol OR ('low density' AND lipoprotein*) OR lipoprotein* OR 'ldl'/exp OR ldl OR 'hdl'/exp OR hdl OR ('high density' AND lipoprotein*) OR (('total'/exp OR total) AND ('cholesterol'/exp OR cholesterol)) OR triglyceride* OR 'lipid'/exp OR lipid OR (('lipid'/exp OR lipid) AND ('metabolism'/exp OR metabolism)) OR antihyperlipidemic

#1. 'alliaceae'/exp OR alliaceae OR 'garlic'/exp OR garlic OR (('garlic'/exp OR garlic) AND extract*) OR 'allium'/exp OR allium OR (('allium'/exp OR allium) AND sativum) OR 'onion'/exp OR onion OR (('onion'/exp OR onion) AND extract*) OR (('allium'/exp OR allium) AND cepa) OR 'brassicaceae'/exp OR brassicaceae OR 'crucifers'/exp OR crucifers OR 'broccoli'/exp OR broccoli OR (('broccoli'/exp OR broccoli) AND extract*) OR 'rocket'/exp OR rocket OR (('rocket'/exp OR rocket) AND ('salad'/exp OR salad)) OR 'cauliflower'/exp OR cauliflower OR (('cauliflower'/exp OR cauliflower) AND extract*)

**Table S1.** Characteristics of the included studies. Abbreviations: ABG, aged black garlic; AGE, aged garlic extract; Apo, apolipoprotein; CAD, coronary artery disease; CHD, coronary heart disease; CKD, chronic kidney disease; FFAs, free fatty acids; HDL-C, high-density lipoprotein cholesterol; LDL-C, low-density lipoprotein cholesterol; Lp(a), lipoprotein a; NEFAs, non-esterified fatty acids; PLs, phospholipids; RCT, randomized controlled trial; SAC, S-allyl cysteine; T2D, type 2 diabetes; TC, total cholesterol; TGs, triglycerides; VLDL-C, very low-density lipoprotein cholesterol. Symbols: * included in the meta-analysis; ~ equivalent to. Values are reported as mg/dl.

| **Study name, study design** | **Study**  **population (inclusion criteria, mg/dl)** | **Definition of dyslipidemia (inclusion criteria)** | **N. of subjects (intervention/ control)** | **Age**  **(years ± SD, range;**  **intervention/control)** | **Intervention/**  **control** | **Daily dosage** | **Period** | **Measured parameters** |
| --- | --- | --- | --- | --- | --- | --- | --- | --- |
| Adler, 1997*, RCT | Subjects with moderate hypercholesterolemia | TC >200 | 12/11 | 45.9 ± 12.6 /  45.4 ± 9.8 | Garlic powder pills (300 mg; Kwai®)/placebo | 900 mg  *(~ 2.7 g of fresh garlic cloves, 35.1 mg alliin, 16.2 mg allicin)* | 12 weeks | TC, LDL-C,  HDL-C, TGs |
| Ashraf, 2005*,  RCT | Patients with T2D and newly diagnosed dyslipidemia | LDL-C >130;  TGs >150;  TC >200;  HDL-C <40 | 33/32 | 60.0 ± 5.0 /  58.0 ± 5.8 | Garlic powder tablets (300 mg; Garlex®)/placebo | 600 mg  *(~ 15.6 mg alliin,*  *7.2 mg allicin)* | 12 weeks | TC, LDL-C,  HDL-C, TGs |
| Aslani, 2016, RCT | Patients with moderate hyperlipidemia | TC 200-240;  LDL-C 100-160 | 27/28 | 45.3 ± 9.3 /  39.3 ± 6.2 | Packets of garlic  (raw garlic)/  no treatment | 20 g | 8 weeks | TC, LDL-C,  HDL-C, TGs |
| Berthold, 1998, randomized crossover trial | Patients with moderate hypercholesterolemia | TC 240-348;  TGs < 265 | -/25 | 58.3 ± 7.5  (total patients) | Steam-distilled garlic oil  (5 mg; Tegra®)/ placebo | 10 mg  *(~ 4-5 g fresh garlic cloves, 4000 U allicin)* | 12 weeks | TC, LDL-C,  HDL-C, TGs |
| Bordia, 1981*, RCT | Patients with CHD and high serum cholesterol | TC 250-330 | 33/29 | 54.7 ± 10.7 /  53.0 ± 11.0 | Garlic essential oil capsules/ placebo | 0.25 mg/kg | 10 months | TC, HDL-C,  LDL-C + VLDL-C, PLs, TC/ PLs |
| Fatima, 2014*, NRCT | Hyperlipidemic patients | - | 55/51 | - | Garlic powder tablets (300 mg; Garlac®)/placebo | 900 mg | 13 weeks | TC, LDL-C,  HDL-C, TGs |
| Feng, 2022, RCT | CKD patients with dyslipidemia | TGs >151;  TC >200;  LDL-C >130 | 30/30 | 52.3 ± 9.0 /  55.2 ± 10.3 | Garlic powder capsules (900 mg; Xuezhitong®) + atorvastatin/  atorvastatin | 2700 mg + 10 mg atorvastatin | 12 weeks | TC, LDL-C,  HDL-C, TGs |
| Gardner, 2007*, RCT | Moderately hypercholesterolemic patients | LDL-C 130-190; TGs < 250 | 42/43 | 49.0 ± 9.0 /  49.0 ± 9.0 | **i)** Raw garlic;  **ii)** garlic powder tablets (Garlicin®);  **iii)** AGE capsules  (300 mg; Kyolic®)/placebo | **i)** 4 g;  **ii)** 4 tablets  *(~ 8% allicin)*;  **iii)** 1.8 g | 24 weeks | LDL-C, HDL-C, TGs, TC/HDL-C |
| Gillingham, 2011, randomized crossover trial | Hypercholesterolemic subjects | LDL-C >116 | 36/- | 47.5 ± 11.9  (total patients) | High-oleic canola oil (Canola Harvest HiLo®) added to cold foods/control diet | Unknown | 26 days | TC, LDL-C,  HDL-C, TGs, TC/HDL-C,  LDL-C/HDL-C, non-HDL-C |
| Heshmat-Ghahdarijani, 2022*,  RCT | Patients  with hyperlipidemia | LDL-C 100-160 | 25/24 | 49.1 ± 8.2 /  48.9 ± 6.1 | Onion powder capsules/  placebo | 1 g | 6 weeks | TC, LDL-C, TGs, HDL-C |
| Isaacsohn, 1998,  RCT | Patients with hypercholesterolemia | LDL-C >160;  TGs < 350 | 28/22 | 58.0 ± 14.0 /  57.0 ± 13.0 | Garlic powder tablets  (300 mg; Kwai®)/  placebo | 900 mg  *(~ 2.7 g of fresh garlic cloves, 35.1 mg alliin, 16.2 mg allicin)* | 12 weeks | TC, LDL-C,  HDL-C, TGs,  Apo A1_,_ Apo B, Lp(a) |
| Jain, 1993*, RCT | Subjects with hypercholesterolemia | TC ≥220 | 20/22 | 48.0 ± 15.0 /  55.0 ± 9.0 | Garlic powder tablets  (300 mg; Kwai®)/  placebo | 900 mg  *(~ 2.7 g of fresh garlic cloves, 35.1 mg alliin, 16.2 mg allicin)* | 12 weeks | TC, LDL-C, TGs, HDL-C |
| Jia, 2020*,  RCT | Patients with hypertriglyceridemia without severe dyslipidemia | TGs 204-576;  LDL-C < 190;  TC < 279 | 141/71 | 51.3 ± 12.6 /  50.6 ± 1.7 | Garlic powder capsules (900 mg; Xuezhitong®)/  placebo | 2700 mg | 12 weeks | TC, LDL-C, TGs, HDL-C |
| Jung, 2014*, RCT | Mildly hypercholesterolemic patients | LDL-C ≥130 | 28/27 | 50.1 ± 9.2 /  50.8 ± 8.1 | Packets of ABG extract (3 g)/  placebo | 6 g  (*~ 600 mg SAC*) | 12 weeks | TC, LDL-C,  HDL-C, TGs, Apo A1_,_ Apo B, FFAs |
| Kannar, 2001*, RCT | Hypercholesterolemic subjects | TC 252-348 | 22/24 | 52.6 ± 10.4 /  57.4 ± 9.0 | Garlic powder tablets (220 mg)/placebo | 880 mg  *(~ 10.4 g fresh garlic, 9.6 mg allicin)* | 12 weeks | TC, LDL-C, TGs, HDL-C,  LDL-C/HDL-C |
| Kojuri, 2007*, RCT | Patients with CAD and newly diagnosed hyperlipidemia | TC ≥200;  LDL-C ≥100 | 50/50 | 55.0 ± 9.8 /  56.5 ± 8.8 | Garlic powder tablets (400 mg)/placebo | 800 mg  *(~ 2 mg allicin)* | 6 weeks | TC, LDL-C, TGs, HDL-C |
| Lash, 1998*, RCT | Renal transplant patients with hypercholesterolemia | TC >240;  LDL-C >160;  TGs < 500 | 19/16 | 43.0 ± 8.7 /  48.0 ± 12.0 | Garlic powder tablets (680 mg; Pure-Gar®)/  placebo | 1360 mg  (*~ 4.1 mg allicin)* | 12 weeks | TC, LDL-C, TGs, HDL-C |
| Lau, 1987  (part I and III),  RCT | Hyperlipidemic patients | TC 220-440; elevated TGs | 25/12 | 45-68  (total patients) | Liquid garlic extract (Kyolic®)/  placebo | 1 g | 24 weeks | TC, TGs |
| Lu, 2015*,  RCT | Subjects with mild hypercholesterolemia | TC ≥200 | 12/11 | 35-55  (total patients) | Yellow onion juice/  placebo | 100 ml | 8 weeks | TC, LDL-C, TGs, HDL-C,  LDL-C/HDL-C |
| Neil, 1996*, RCT | Patients with moderate hyperlipidaemia | TC 232-329;  LDL-C ≥135; TG ≤ 496;  HDL-C ≤ 77 | 57/58 | 53.0 ± 7.0 /  52.6 ± 7.8 | Garlic powder tablets (300 mg; Kwai®)/placebo | 900 mg  *(~ 2.7 g of fresh garlic cloves, 35.1 mg alliin, 16.2 mg allicin)* | 24 weeks | TC, LDL-C,  HDL-C, TGs,  Apo A1_,_ Apo B |
| Peleg, 2003*, RCT | Patients with mild to moderate primary hypercholesterolemia | LDL-C 130-190; TGs < 400 | 13/20 | 52.4 ± 7.5 /  54.7 ± 7.5 | Garlic powder tablets (Inodiel®)/ placebo | 4 tablets  (*~ 22.4 g alliin)* | 16 weeks | TC, LDL-C, TGs, HDL-C |
| Satitvipawee, 2003*,  RCT | Hypercholesterolemic subjects | TC ≥200 | 70/66 | 47.0 ± 6.6 /  47.0 ± 6.0 | Garlic powder tablets (333 mg)/ placebo | 333 mg  (*~ 7 g fresh garlic,*  *5.6 mg allicin)* | 12 weeks | TC, LDL-C, TGs, HDL-C |
| Simons, 1995, randomized crossover trial | Subjects with mild to moderate hypercholesterolemia | TC 213-312;  TG < 266 | 28/28 | 53.6 ± 10.4  (total patients) | Garlic powder tablets (300 mg; Kwai®)/placebo | 900 mg  *(~ 2.7 g of fresh garlic cloves, 35.1 mg alliin, 16.2 mg allicin)* | 12 weeks | TC, LDL-C, TGs, HDL-C, Lp(a), lathosterol/TC, LDL receptor mass, LDL oxidation rate |
| Sobenin, 2008*, RCT | Men with mild hypercholesterolemia | TC 224-271;  LDL-C 135-178;  HDL-C 25-75 | 23/19 | 51.7 ± 9.6 /  51.7 ± 10.9 | Garlic powder tablets (300 mg; Allicor®)/placebo | 600 mg  (*~ 15.6 mg allicin)* | 12 weeks | TC, LDL-C, TGs, HDL-C |
| Sobenin, 2010*, RCT | Patients with CHD and hypercholesterolemia | TC >200 | 26/25 | 56.7 ± 9.2 /  56.3 ± 8.5 | Garlic powder tablets (150 mg; Allicor®)/placebo | 300 mg  (*~ 3.9 mg allicin)* | 12 months | TC, LDL-C, TGs, HDL-C |
| Steiner, 1996, randomized crossover trial | Moderately hypercholesterolemic men | TC 220-290 | 41/41 | 32-68  (total patients) | AGE capsules  (800 mg; Kyolic®)/placebo | 7.2 g | 24 weeks | TC, LDL-C, TGs, HDL-C |
| Superko, 2000*, RCT | Moderately hypercholesterolemic subjects | LDL-C 150-200; TGs < 300 | 25/25 | 53.0 ± 10.0  (total patients) | Garlic powder tablets (300 mg; Kwai®)/placebo | 900 mg  *(~ 2.7 g of fresh garlic cloves, 35.1 mg alliin, 16.2 mg allicin)* | 12 weeks | TC, LDL-C, TGs, HDL-C, Apo B, Lp(a), LDL diameter |
| Valls, 2022*, randomized crossover trial | Patients with moderate hypercholesterolemia | LDL-C 115-190 | 62/65 | 53.2 ± 8.4 | ABG powder tablets (250 mg)/placebo | 250 mg  *(~1.25 mg SAC,*  *0.1 mg alliin)* | 6 weeks | TC, LDL-C, TGs, HDL-C, TC/HDL-C,  LDL-C/HDL-C,  Apo A1, Apo B,  Apo B/Apo A1,  TGs/Apo B, NEFAs |

*
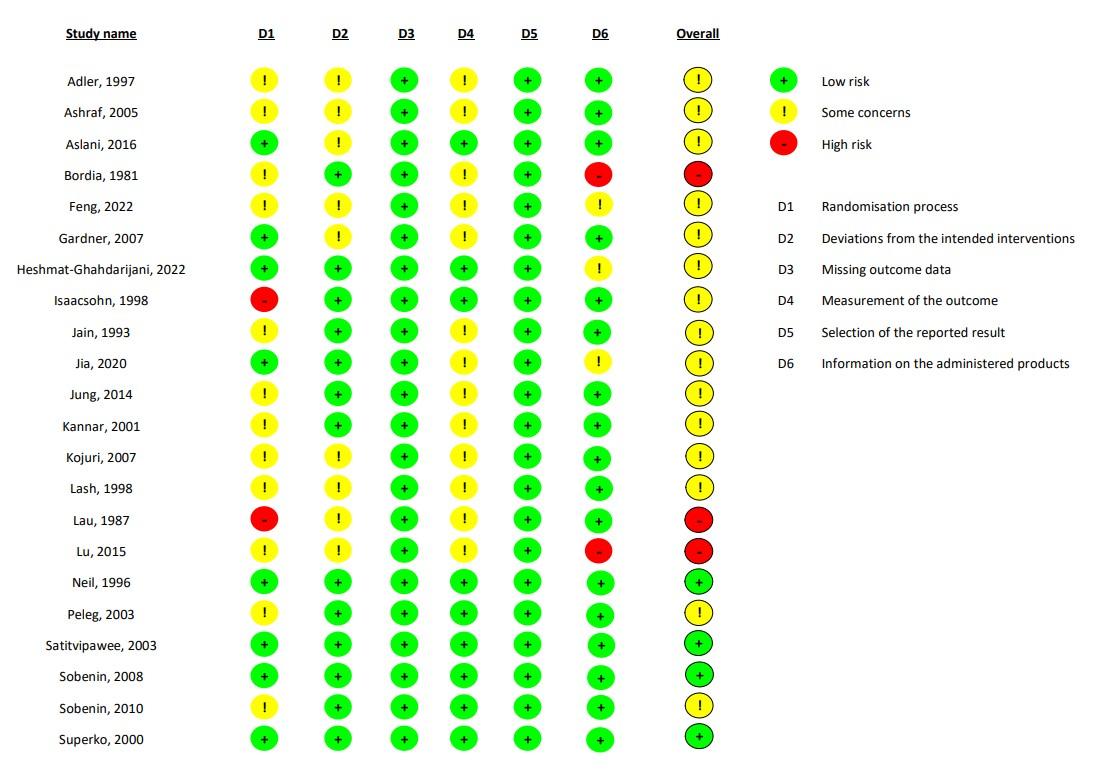
*

**Figure S1.** Risk of bias of the included randomized controlled trials. Legend: green circle, low risk; yellow circle, moderate risk; red circle, high risk.


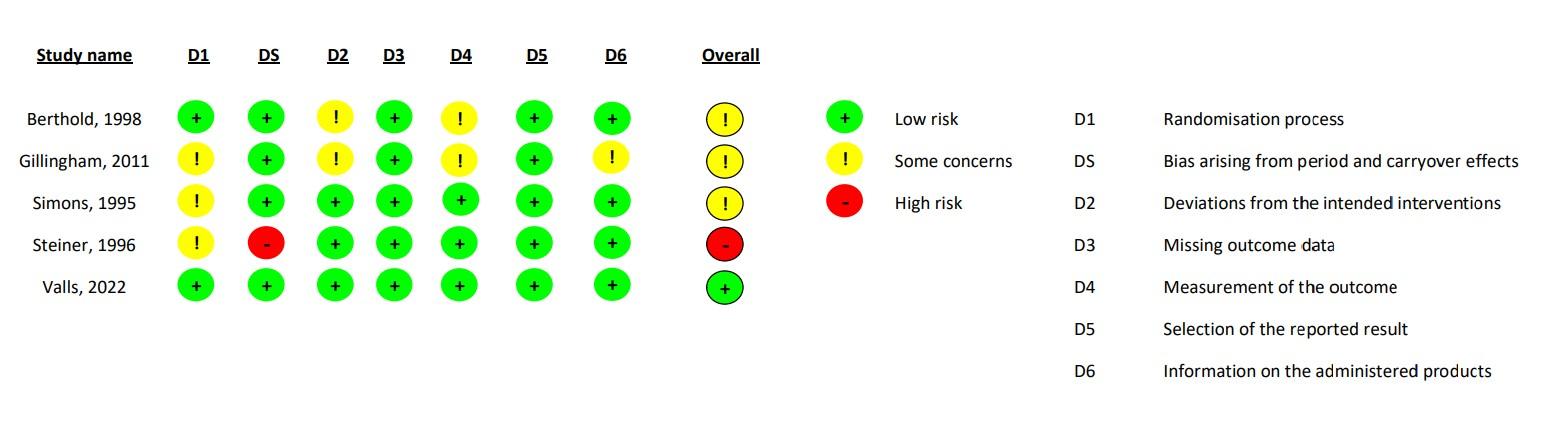


**Figure S2.** Risk of bias of the included randomized crossover trials. Legend: green circle, low risk; yellow circle, moderate risk; red circle, high risk.


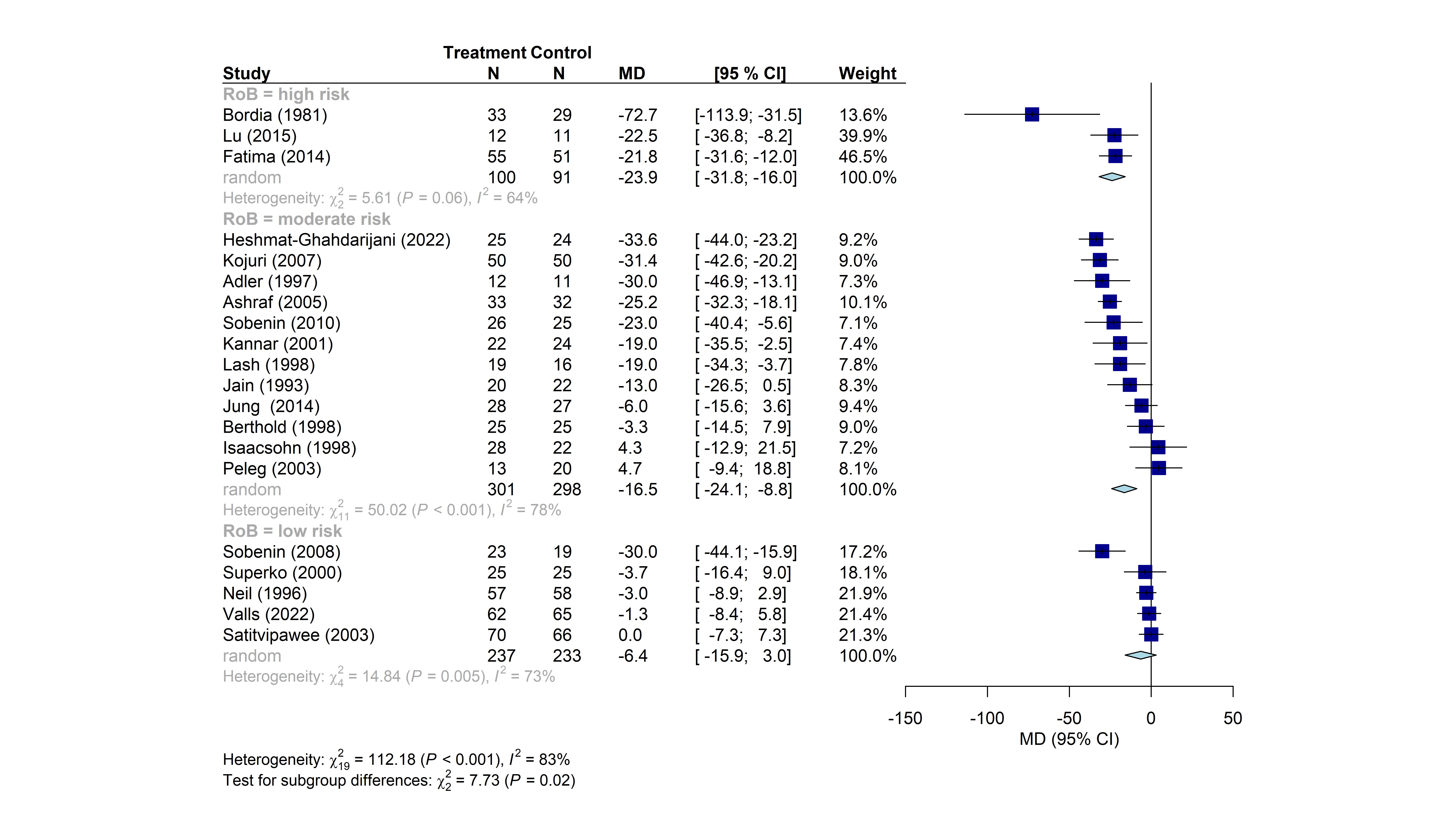


**Figure S3.** Forest plot of the mean difference (MD) of total cholesterol (TC) levels (mg/dl) with 95% Confidence Interval (CI) in patients receiving *Alliaceae* extracts (treatment group) compared with the control group (placebo/no treatment) stratified by risk of bias.


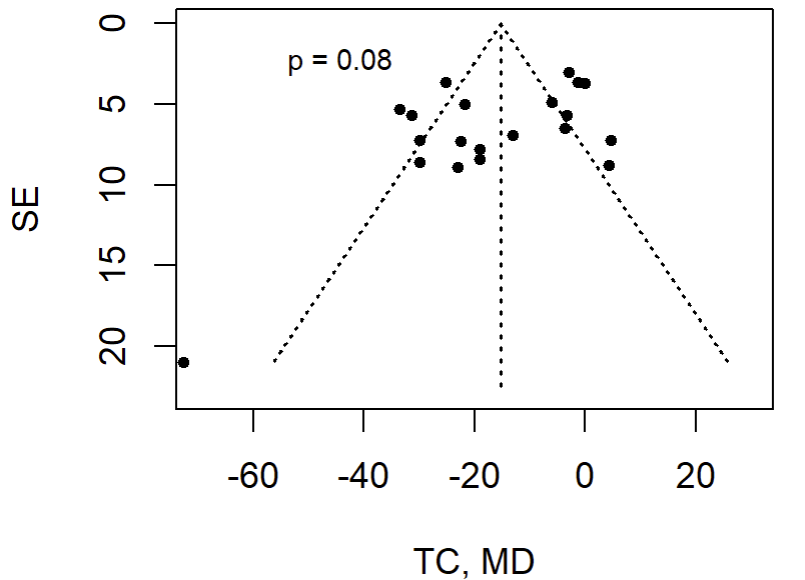


**Figure S4**. Funnel plot of total cholesterol (TC) showing the mean difference (MD) and standard error (SE) for each study. The p-value of Egger’s test is also reported.


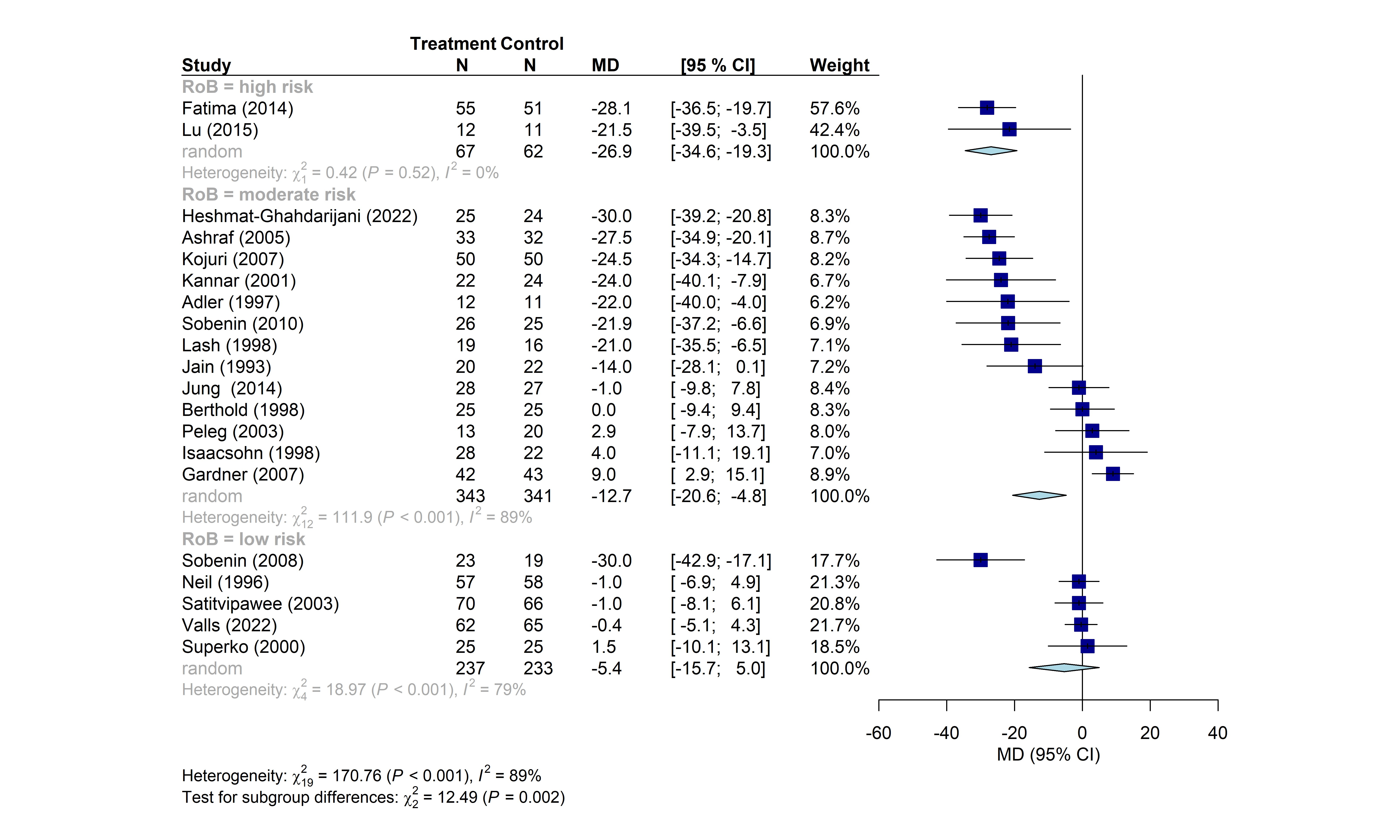


**Figure S5**. Forest plot of the mean difference (MD) of low-density lipoprotein cholesterol (LDL-C) levels (mg/dl) with 95% Confidence Interval (CI) in patients receiving *Alliaceae* extracts (treatment group) compared with the control group (placebo/no treatment) stratified by risk of bias.


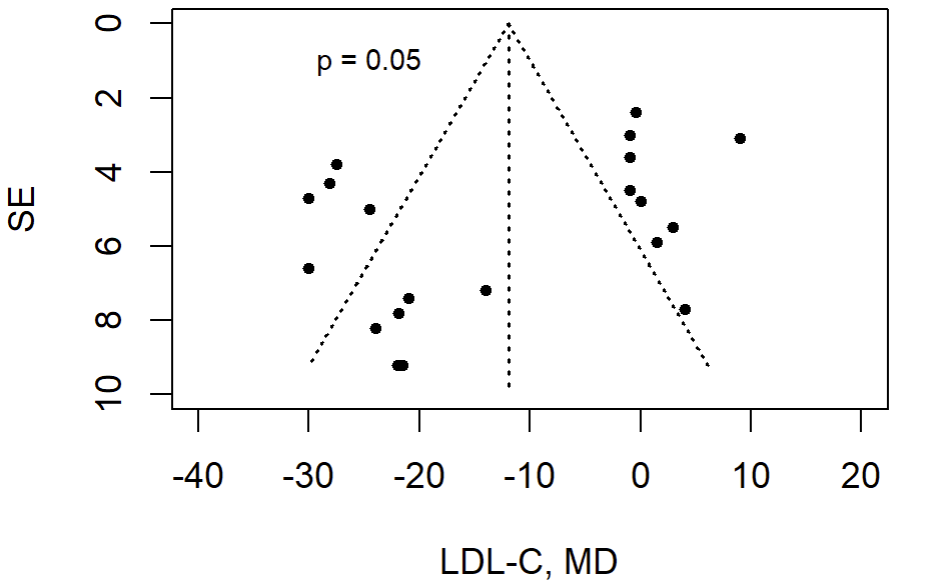


**Figure S6**. Funnel plot of low-density lipoprotein cholesterol (LDL-C) showing the mean difference (MD) and standard error (SE) for each study. The p-value of Egger’s test is also reported.


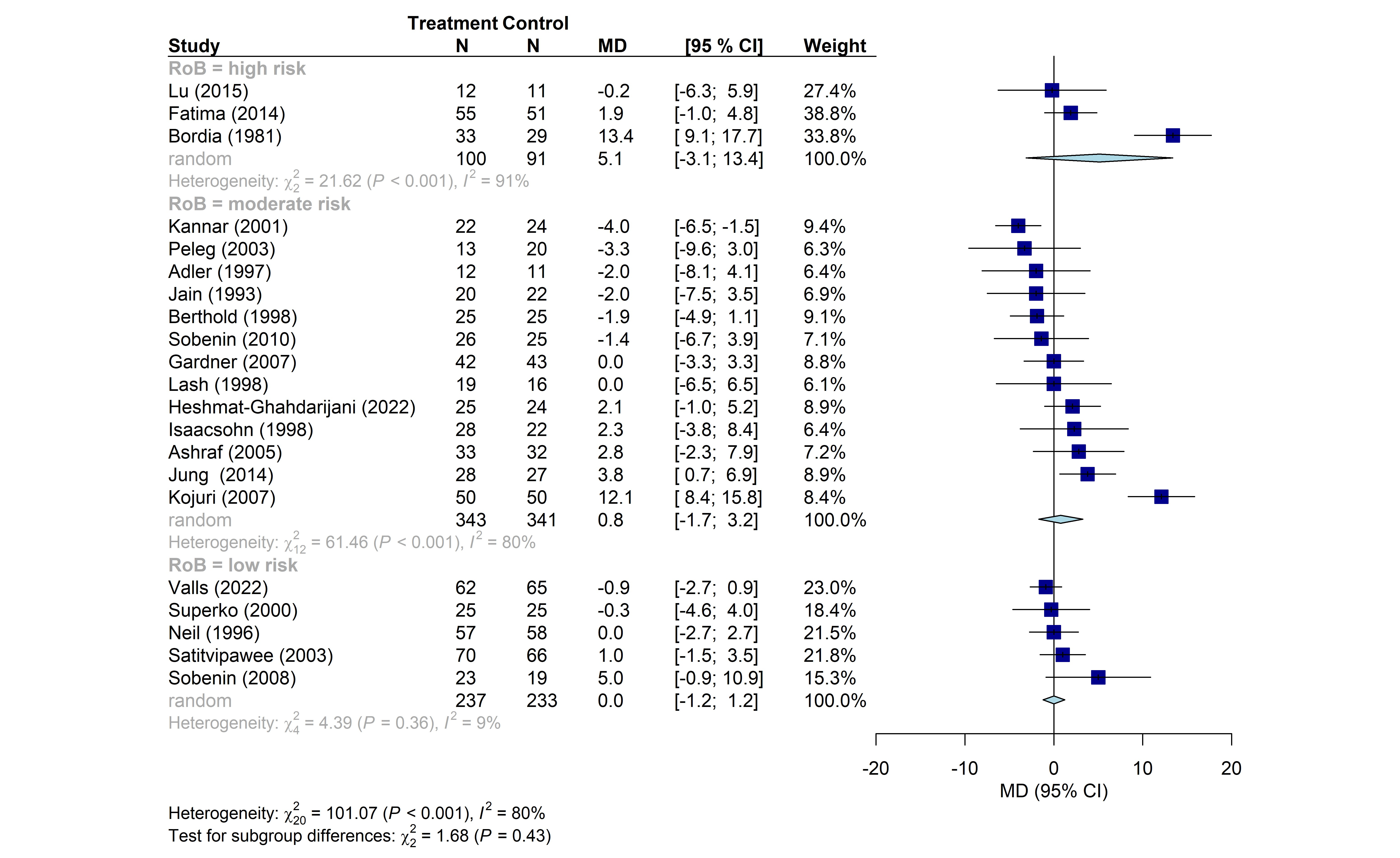


**Figure S7.** Forest plot of the mean difference (MD) of high-density lipoprotein cholesterol (HDL-C) levels (mg/dl) with 95% Confidence Interval (CI) in patients receiving *Alliaceae* extracts (treatment group) compared with the control group (placebo/no treatment) stratified by risk of bias.


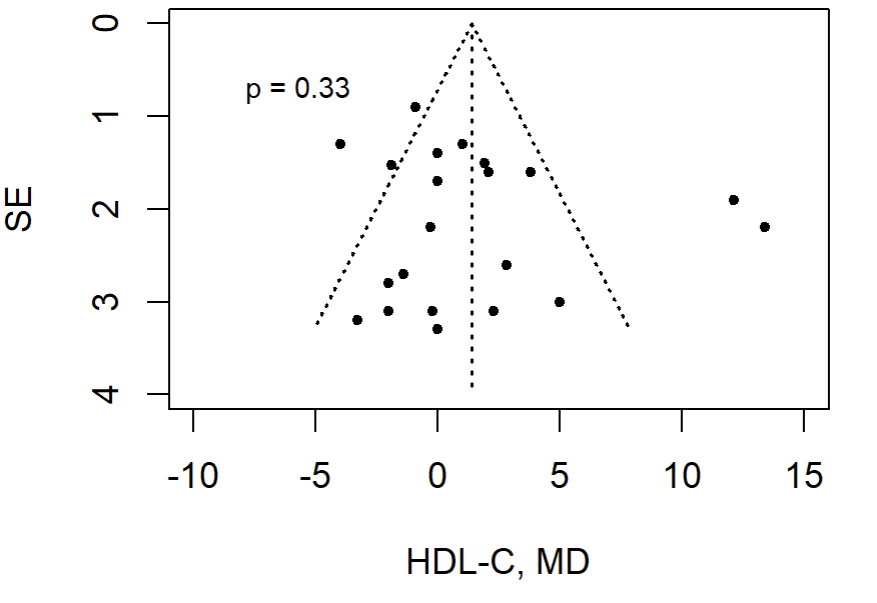


**Figure S8**. Funnel plot of high-density lipoprotein cholesterol (HDL-C) showing the mean difference (MD) and standard error (SE) for each study. The p-value of Egger’s test is also reported.


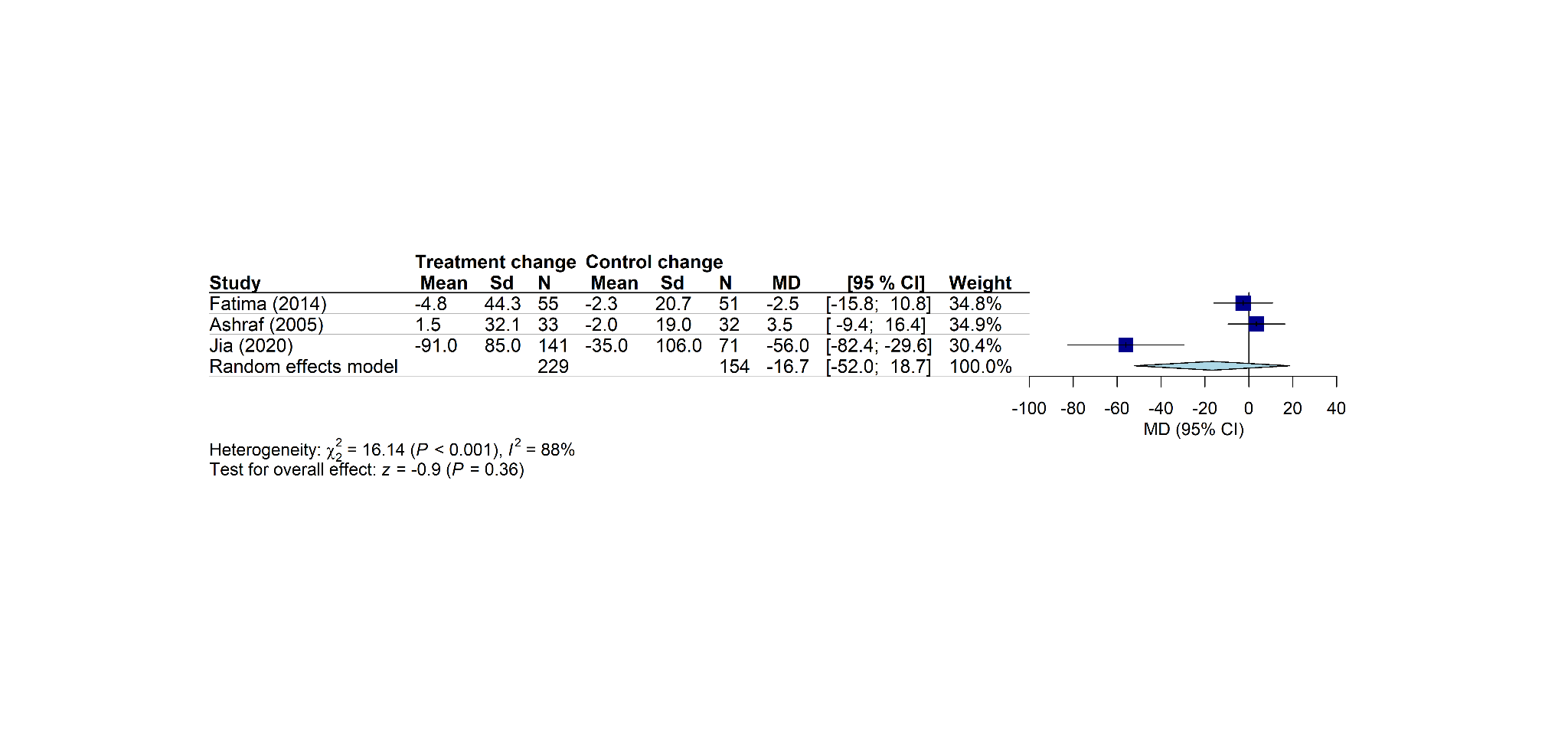


**Figure S9.** Forest plot of the mean difference (MD) of triglycerides (TGs) levels (mg/dl) with 95% Confidence Interval (CI) in patients receiving garlic extracts (treatment group) compared with the control group (placebo).


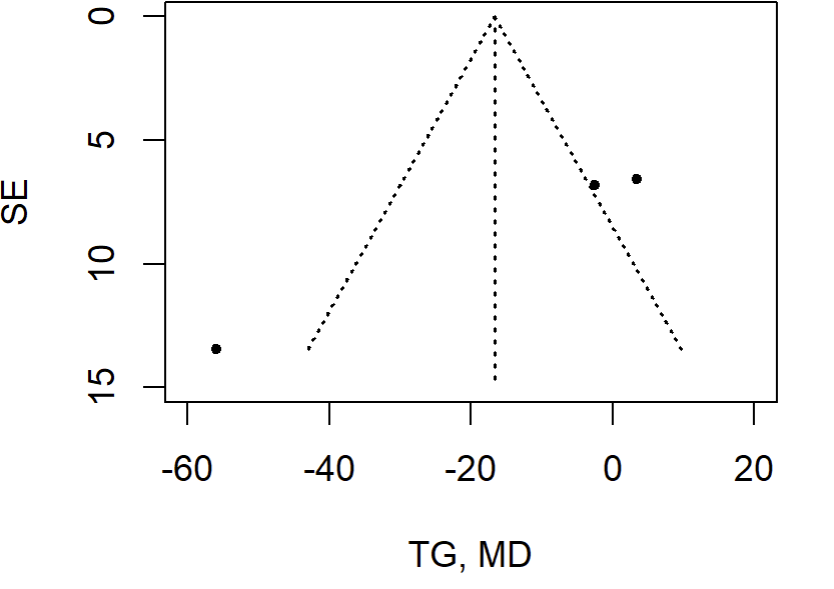


**Figure S10**. Funnel plot of triglycerides (TGs) showing the mean difference (MD) and standard error (SE) for each study.

**Table S2. Summary of findings table: results of the GRADE assessment.** Abbreviations: TC: total cholesterol; LDL-C: low-density lipoprotein cholesterol; HDL-C: high-density lipoprotein cholesterol; TGs: triglycerides; CI: confidence interval; MD: mean difference. Explanations: **^a^** Fifteen out of 20 studies had moderate (12) or high (3) risk of bias. Meta-analysis stratified by risk of bias showed that the overall effect size was driven by these studies, as studies with low risk of bias (5) did not show a significant effect of *Alliaceae* extracts on TC levels. **^b^** There was no overlap in some confidence intervals, the p-value for heterogeneity was less than 0.05, and the I^2^ was large (83.0%). Heterogeneity could be explained by differences in the methodological quality of studies and interventions. **^c^** There was no overlap in some confidence intervals, the p-value for heterogeneity was less than 0.05, and the I^2^ was large (89.0%). Heterogeneity could be explained by differences in the methodological quality of studies and interventions. **^d^** There was no overlap in some confidence intervals, the p-value for heterogeneity was less than 0.05, and the I^2^ was large (80.0%). Heterogeneity could be explained by differences between interventions. **^e^** The 95% CI included a mean difference of 0. **^f^** All studies had moderate or high risk of bias. **^g^** There was no overlap in some confidence intervals, the p-value for heterogeneity was less than 0.05, and the I^2^ was large (88.0%). Heterogeneity could be explained by the low number of studies, as well as differences in the methodological quality of studies and interventions. **^h^** The number of studies was very low and, for one study, no information on baseline TG levels was available.

| **Certainty assessment** | | | | | | | **N. of patients** | | **Effect** | **Certainty** | **Importance** |
| --- | --- | --- | --- | --- | --- | --- | --- | --- | --- | --- | --- |
| **N. of studies** | **Study design** | **Risk of bias** | **Inconsistency** | **Indirectness** | **Imprecision** | **Other considerations** | ***Alliaceae* extracts** | **Placebo/ No treatment** | **Absolute (95% CI)** |  |  |
| **TC (follow-up: 12 weeks, median)** | |  |  |  |  |  |  |  |  |  |  |
| 20 | Randomized trials: 19/20  Non-randomized trials: 1/20 | Very serious^a^ | Serious^b^ | Not serious^a^ | Not serious^a^ | Strong association | 638 | 622 | MD **15.2 mg/dl lower** (21.3 lower to 9.1 lower) | ⨁⨁◯◯ Low | CRITICAL |
| **LDL-C (follow-up: 12 weeks, median)** | |  |  |  |  |  |  |  |  |  |  |
| 20 | Randomized trials: 19/20  Non- randomized trials: 1/20 | Very serious^a^ | Serious^c^ | Not serious | Not serious | Strong association | 647 | 636 | MD **11.9 mg/dl lower** (18.1 lower to 5.8 lower) | ⨁⨁◯◯ Low | CRITICAL |
| **HDL-C (follow-up: 12 weeks, median)** | |  |  |  |  |  |  |  |  |  |  |
| 21 | Randomized trials: 21/21 | Not serious | Serious^d^ | Not serious | Serious^e^ | None | 680 | 665 | MD **1.4 mg/dl higher** (0.5 lower to 3.3 higher) | ⨁⨁◯◯ Low | IMPORTANT |
| **TGs (follow-up: 12 weeks, median)** | |  |  |  |  |  |  |  |  |  |  |
| 3 | Randomized trials: 3/3 | Very serious^f^ | Serious^g^ | Serious^h^ | Serious^e^ | Strong association | 229 | 154 | MD **16.7 mg/dl lower** (52 lower to 18.7 higher) | ⨁◯◯◯ Very low | CRITICAL |
